# Module walking using an SH3-like cell-wall-binding domain leads to a new GH184 family of muramidases

**DOI:** 10.1107/S2059798323005004

**Published:** 2023-07-10

**Authors:** Olga V. Moroz, Elena Blagova, Andrey A. Lebedev, Lars K. Skov, Roland A. Pache, Kirk M. Schnorr, Lars Kiemer, Esben P. Friis, Søren Nymand-Grarup, Li Ming, Liu Ye, Mikkel Klausen, Marianne T. Cohn, Esben G. W. Schmidt, Gideon J. Davies, Keith S. Wilson

**Affiliations:** a York Structural Biology Laboratory, Department of Chemistry, University of York, York YO10 5DD, United Kingdom; b CCP4, STFC Rutherford Appleton Laboratory, Harwell Oxford, Didcot OX11 0QX, United Kingdom; c Novozymes A/S, Biologiens Vej 2, 2800 Kgs Lyngby, Denmark; d Novozymes Investment Co. Ltd, 14 Xinxi Road, Beijing 100085, People’s Republic of China; University of Cambridge, United Kingdom

**Keywords:** GH184 family, lysozymes, lysins, peptidoglycan cleavage, SH3-like domains, muramidases, glycoside hydrolase family 24, *Trichophaea saccata*, module walking

## Abstract

Muramidases (also known as lysozymes) hydrolyse the peptidoglycan component of the bacterial cell wall and are found in many glycoside hydrolase (GH) families. Similar to other glycoside hydrolases, muramidases sometimes have noncatalytic domains that facilitate their interaction with the substrate. Here, the identification, characterization and X-ray structure of a novel fungal GH24 muramidase from *Trichophaea saccata* is first described, in which an SH3-like cell-wall-binding domain (CWBD) was identified by structure comparison in addition to its catalytic domain. Further, a complex between a triglycine peptide and the CWBD from *T. saccata* is presented that shows a possible anchor point of the peptidoglycan on the CWBD. A ‘domain-walking’ approach, searching for other sequences with a domain of unknown function appended to the CWBD, was then used to identify a group of fungal muramidases that also contain homologous SH3-like cell-wall-binding modules, the catalytic domains of which define a new GH family. The properties of some representative members of this family are described as well as X-ray structures of the independent catalytic and SH3-like domains of the *Kionochaeta* sp., *Thermothielavioides terrestris* and *Penicillium virgatum* enzymes. This work confirms the power of the module-walking approach, extends the library of known GH families and adds a new noncatalytic module to the muramidase arsenal.

## Abstract 2

The identification, characterization and X-ray structure of a novel fungal GH24 muramidase from *Trichophaea saccata* is described in which an SH3-like cell-wall-binding domain was identified by structure comparisons in addition to its catalytic domain. A domain-walking approach was then used to identify a group of fungal muramidases that belong to a new GH family containing homologous SH3-like cell-wall-binding modules, and X-ray structures of the independent catalytic and SH3-like domains of three of them are reported.

## 1. Introduction

Muramidases are *N*-acetylmuramide glycanhydrolases which cleave the β-1,4-glycosidic bond between *N*-acetylmuramic acid (NAM) and *N*-acetylglucosamine (NAG) in the carbohydrate backbone of the bacterial cell-wall peptidoglycan. They were previously known as lysozymes, a name which is still in common use. The first lysozyme was discovered serendipitously by Fleming, who observed antibacterial action when he treated bacterial cultures with nasal mucus from a patient suffering from a cold and named the enzyme ‘lysozyme’ (Fleming, 1922 ▸). Fleming showed that there were similar enzymes in a wide range of organisms, including the hen *Gallus gallus*, with hen egg-white lysozyme (HEWL) being one of the most extensively studied enzymes and the first for which a 3D structure was determined (Blake *et al.*, 1962 ▸, 1965 ▸). These lysozymes were later classified as members of glycoside hydrolase family 22 (GH22) in the Carbohydrate Active Enzymes database (CAZy; http://www.cazy.org/; Lombard *et al.*, 2014 ▸; CAZypedia Consortium, 2018 ▸). The number EC 3.2.1.17 was assigned to these proteins by The Enzyme Commission, who also recommended that the name lysozyme be replaced by muramidase or *N*-acetylmuramide glycanohydrolase (International Union of Biochemistry, 1961 ▸). We will use the name muramidase throughout. Muram­idase activity has now been found in several CAZy GH families: GH18, GH19, GH22, GH23, GH24, GH25, GH73 and GH108. The muramidases in the various families cleave the same substrate, but do so via a number of mechanisms. A number of glycoside hydrolases, including some muramidases, have extra domains in addition to their catalytic domains. Many of these are carbohydrate-binding modules (CBMs), which target the enzymes to their saccharide substrate, facilitate binding and disrupt insoluble substrate fractions (Sidar *et al.*, 2020 ▸). At present there are 88 CBM families in the CAZy database (http://www.cazy.org/).

In cell-wall hydrolases, the additional modules can be broadly classified as cell-wall-binding domains (CWBDs) that differ depending on the component of the cell wall to which they bind (Vermassen *et al.*, 2019 ▸). One example of a CWBD is the SH3 [sarcoma (src) homology 3] domain (Mayer *et al.*, 1988 ▸) that can be located in the N- or C-terminal regions of such hydrolases. SH3 domains consist of five to eight β-strands forming two orthogonal antiparallel β-sheets (Kurochkina & Guha, 2013 ▸). The classical SH3 domains are defined in SCOPe (Structural Classification of Proteins – extended; Fox *et al.*, 2014 ▸; Chandonia *et al.*, 2022 ▸) as Fold b.34: SH3-like β-barrel, partly opened, with the last strand interrupted by a turn of 3_10_-helix. Classical SH3 domains are responsible for regulating protein–protein interactions in signal transduction pathways (Schlessinger, 1994 ▸).

The bacterial SH3 homology domains were identified later than their eukaryotic counterparts by comparative genomics approaches (Ponting *et al.*, 1999 ▸; Whisstock & Lesk, 1999 ▸), and it was suggested that their functions differ from those of the eukaryotic domains based on sequence analysis. It was suggested that an early horizontal gene transfer could have occurred between eukaryotes and bacteria, with the direction of transfer still unclear. Ponting and coworkers suggested that these domains could originally have evolved in bacteria and have been transferred to eukaryotes as a result of mitochondrial endosymbiosis, but other possibilities were not excluded. These domains are annotated as SH3-like or SH3b domains [PDOC51781 in PROSITE (Sigrist *et al.*, 2013 ▸), PF08460 in Pfam, now InterPro (Chandonia *et al.*, 2022 ▸)]; the name SH3b was suggested and three-dimensional structures were reviewed by Kamitori & Yoshida (2015 ▸). It has been hypothesized that they play a crucial role in recognizing and binding to bacterial cell walls, serving as targeting domains (Chang & Ryu, 2017 ▸). For several phage endolysins it has been demonstrated that the SH3 domain is required for optimal activity; for example, an approximately tenfold reduction of activity was reported for PlyTW phage Twort endolysin in the absence of its SH3 domain (Becker *et al.*, 2015 ▸). Further discussion of SH3 domains will follow in Section 3.

Screening for new enzymes with muramidase activity with potential benefits for industrial application in poultry feeds, where the enzymes can degrade bacterial cell-wall residues, previously led to the identification of the first commercial product, a GH25 enzyme from *Sodiomyces alcalophilus* marketed as Balancius^TM^ (Moroz *et al.*, 2021 ▸; Li *et al.*, 2018 ▸). The screening project not only included GH25 muramidases but also other muramidase families known to be present in fungal taxa at the time: GH23 and GH24. Here, we describe how this screening has now led to the discovery of a fungal GH24 muramidase from *Trichophaea saccata* with an SH3-like CWBD attached to the catalytic domain, often called the core domain (CD). The domain structure is described in a publicly available patent (Liu *et al.*, 2017 ▸). The evolution of the GH24 muramidases has been extensively analysed in terms of coopting a toxic phage gene for a core cellular function in a large bacterial clade (Randich *et al.*, 2019 ▸). Here, we report the identification of several new fungal GH24s with this CWBD and the structure of the intact *T. saccata* enzyme, henceforth referred to as *Ts*CWBD-GH24.

In order to identify additional catalytic domains associated with this SH3-like module, a ‘module-walking’ approach was used. Module walking is an inventive discovery tool based on the observation that diverse catalytic functions (hydrolases, esterases, lyases, oxidases, phosphorylases *etc.*) often share similar binding modules that target the catalytic modules to a given, often polymeric, substrate; working on the basis that ‘the friends of my friends are my friends’, new modules and new catalytic entities can be identified for subsequent functional and structural analysis. For example, for the discovery of a new family of chitin-active lytic polysaccharide monooxygenases (LPMOs), Hemsworth and coworkers used the knowledge of a common putative chitin-binding domain observed in GH18 chitinases (Hemsworth *et al.*, 2014 ▸). Here, the presence of an SH3-like CWBD was used to search for previously uncharacterized domains sharing the SH3-like CWBD. The module was thus used to search sequence databases, resulting in the discovery of a new family of muramidases which has been assigned the CAZy number GH184. Here, we describe the identification of a significant number of fungal members of this family and present three-dimensional structures of individual catalytic or SH3-like domains from three different fungal species, *Kionochaeta* sp., *Thermothielavioides terrestris* and *Penicillium virgatum*, henceforth named *Ks*GH184, *Tt*GH184 and *Pv*GH184, respectively.

## 2. Materials and methods

### 2.1. Screening for new muramidases identifies a fungal GH24 with an extra domain

*T. saccata* CBS804.70 was purchased from the Centraalbureau voor Schimmelcultures (Utrecht, The Netherlands). The strain was originally isolated in Staffordshire, England from coal-contaminated soil with high surface temperatures. It was clear from the amino-acid sequence of the GH24 muramidase (NCBI ID ON783686) that this enzyme contained an extra N-terminal domain. In this study, the full-length GH24 enzyme (*Ts*CWBD-GH24) and two truncated versions corresponding to the individual domains, *Ts*GH24-CD and *Ts*CWBD, were expressed and examined.

### 2.2. Discovery of other GH24s/GH184s with a CWBD

The putative CWBD was extracted from the full-length *T. saccata* GH24 sequence and used to seed a *BLAST* search for similar occurrences in other sequences (both Novozymes and public sequence databases were used). The ∼500 identified hits were aligned with *MUSCLE* (Edgar, 2004 ▸) and the alignment was inspected manually to weed out incorrect matches using criteria such as cysteine patterns and incorrect gene models. The final curated alignment was used to create a sensitive hidden Markov model (HMM) using *HMMER* 3.0 (Eddy, 2011 ▸). The hits picked for expression were confirmed by the HMM model. Details of the HMM model can be found in patents (Liu *et al.*, 2017 ▸, 2018 ▸).

#### 2.2.1. Module walking with the SH3-like CWBD: a new GH184 muramidase family

Using the CWBD from the *T. saccata* GH24 enzyme, a *BLAST* search of Novozymes and external databases was performed and led to the identification of a number of genes coding for enzymes containing homologous domains. The reading frame of one of these sets of enzymes had no previous annotation and included a CWBD at the N-terminus of the protein, the same configuration as in the *Ts*GH24 enzyme. The amino-acid sequences of this set of proteins did not fit into any of the current GH families. They had common sequence features (HMMs) and therefore were suggested to belong to a new GH family, GH184. Based on these results, a selection of GH184s were targeted for expression, purification and characterization. The novel CWBD was later identified as an SH3-like domain using structural comparisons with *GESAMT* (Krissinel, 2012 ▸) after the X-ray structure had been determined, as described below.

### 2.3. Cloning, expression and purification of GH24 and GH184 muramidases

The new GH24/GH184 muramidases with a CWBD were cloned and expressed by established protocols (Liu *et al.*, 2017 ▸, 2018 ▸). Unless otherwise stated, all chemicals/reagents were purchased from Sigma–Aldrich and were reagent grade. Purifications were carried out by standard techniques, typically involving cation or anion exchange. As examples of the procedures, details of the cloning, expression and purification of *Ts*CWBD-GH24 and *Tt*GH184 can be found in the supporting information. GenBank entries for the proteins studied here can be found in Tables 3 and 5 and Supplementary Table S1. An E41A mutant of *Ks*GH184 was produced and purified using the same methods as used for *Ks*GH184.

### 2.4. Evidence for muramidase activity

Muramidase activity on peptidoglycan was measured using the turbidity (the OD-drop assay) and reducing-ends assays detailed below.

#### 2.4.1. Preparation of peptidoglycan for assays

Lyophilized cells of *Micrococcus lysodeikticus* ATTC No. 4698 were obtained from Sigma–Aldrich (catalogue No. M3770) and were used as the peptidoglycan substrate in the assays. *M. lysodeikticus* has been renamed *M. luteus* (Benecky *et al.*, 1993 ▸), but here we will use the commercial name.

#### 2.4.2. Activity assay by reduction in turbidity (the OD-drop assay)

The OD-drop assay measures muramidase/lysozyme activity through the reduction in optical density (OD) caused by turbidity (light scattering), as described in many papers on HEWL (Parry *et al.*, 1965 ▸; Dobson *et al.*, 1984 ▸). Enzyme activities at 37°C were determined by measuring the decrease (drop) in the optical density of a solution of resuspended *M. lysodeikticus* ATTC No. 4698 using a Tecan Infinite M200 reader at 540 nm (Shugar, 1952 ▸; https://www.sigmaaldrich.com/technical-documents/protocols/biology/enzymatic-assay-of-lysozyme.html). Before use, the *M. lysodeikticus* cells were resuspended to a concentration of 0.5 mg ml^−1^ in citric acid/phosphate buffer pH 6.0 and the OD at 540 nm was measured. The cell suspension was adjusted so that the cell concentration equalled an OD_540_ of approximately 1 and the adjusted cell suspension was stored at 4°C before use. Resuspended cells were used within 4 h. The values are the averages of at least four determinations of the reduction in OD_540_ after 60 min reaction time.

#### 2.4.3. Activity on peptidoglycan at pH 5.0 using a reducing-ends assay

When peptidoglycan is hydrolysed by a muramidase, new saccharide reducing ends (aldehyde groups) are produced and the increase in reducing ends can be used as a measure of glycolytic activity. After incubation and further acid hydrolysis of soluble carbohydrate oligosaccharides, the amount of reducing ends produced was determined by reaction with *para*-hydroxybenzoic acid hydrazide. The resulting hydrazone has a yellow colour and can be detected at 405 nm.

The muramidases were diluted in citrate/phosphate dilution buffer (5 m*M* sodium citrate, 5 m*M* K_2_HPO_4_, 0.01% Triton X-100 pH 5.0) to 200 or 50 µg ml^−1^ in polypropylene tubes, dependent on the concentrations of the available stock solutions. The solutions were further diluted in a 96-well polypropylene microtitre plate by preparing a twofold dilution series down to a concentration of 4.0 µg ml^−1^ in phosphate dilution buffer. The muramidase concentration in the assay is ten times lower after mixing with the substrate (see below). The assay can be performed with peptidoglycan from several sources; we describe it below using *M. lysodeikticus* as an example.

A 50 mg ml^−1^ stock solution of *M. lysodeikticus* substrate in water was prepared and diluted to 250 µg ml^−1^ in citrate/phosphate buffer (50 m*M* sodium citrate, 50 m*M* K_2_HPO_4_ pH 5.0). In a polypropylene deep-well plate, 50 µl of the muramidase dilution was mixed with 450 µl *M. lysodeikticus* solution and incubated at 40°C with shaking (500 rev min^−1^) for 45 min. After incubation, the deep-well plates were centrifuged (3200 rev min^−1^, 7 min) to pellet insoluble material and 100 µl of the supernatant was mixed with 50 µl 3.2 *M* HCl in a 96-well PCR plate and incubated at 95°C for 80 min. 50 µl 3.5 *M* NaOH was added to each well of the PCR plate and 150 µl of each sample was transferred to a new PCR plate containing 75 µl 4-hydroxybenzhydrazide (PAHBAH) solution in potassium/sodium tartrate/NaOH buffer (50 g l^−1^ potassium/sodium tartrate + 20 g l^−1^ NaOH) per well. The plate was incubated at 95°C for 10 min before 100 µl samples were transferred into a clear flat-bottomed microtitre plate for optical density (OD) measurements at 405 nm and 25°C. OD measurements were also performed on threefold-diluted samples (50 µl sample diluted in 100 µl Milli-Q water at 25°C) to ensure a reading in the linear range. The OD measurement values shown in Tables 3 and 5 represent the difference after the original (background) reading had been subtracted and are the average of two OD measurement values.

### 2.5. Evidence for bacterial cell-wall binding by the *T. saccata* CWBD

The following is directly based on the published patent (Liu *et al.*, 2017 ▸), in which it is shown that the *T. saccata* CWBD binds to bacterial cells. The procedure was as follows. 250 mg *M. lysodeikticus* ATCC No. 4698 cells were resuspended in 2.5 ml H_2_O with 0.1% Tween 80. The cells were treated at 4°C overnight. Avicel PH-101 is a microcrystalline cellulose powder trademarked by FMC Corporation (Philadelphia, Pennsylvania, USA) and sold by Sigma–Aldrich (catalogue No. 11365). 250 mg Avicel was suspended in H_2_O with 0.1% Tween 80. This was also left to hydrate overnight.

After overnight hydration, 50 µl of each suspension was removed and washed once in 50 µl H_2_O with 0.1% Tween 80. The purified *Ts*CWBD had a concentration of 0.23 mg ml^−1^ in a buffer consisting of 50 m*M* sodium acetate pH 4.5, 50 m*M* NaCl. For the experiment, 50 µl Avicel suspension or 50 µl *M. lysodeikticus* suspension were aliquoted into 1.5 ml Eppendorf tubes. 50 µl (11.5 mg) of purified *Ts*CWBD protein was then added to each tube, mixed by vortexing and incubated at room temperature for 30 min. The samples were then centrifuged and the liquid was decanted into a 1.5 ml Eppendorf tube.

**Figure 1:**
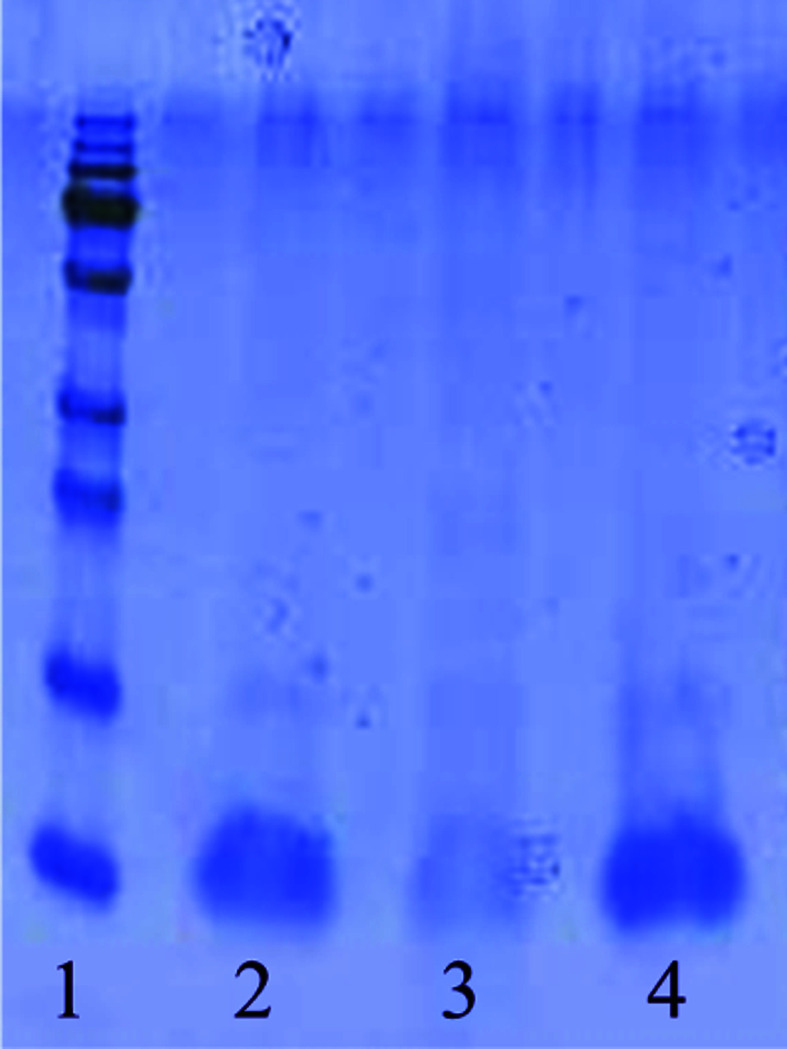
SDS–PAGE analysis of *Ts*CWBD in the supernatant after incubation with Avicel (control) or *M. lysodiekticus* cells shows a reduction in the *Ts*CWBD content in the supernatant after incubation with *M. lysodiekticus* cells. Lane 1, molecular-weight marker (from the bottom: 10, 15, 25, 35, 55, 70, 100, 130 and 250 kDa); lane 2, *Ts*CWBD incubated with Avicel; lane 3, *Ts*CWBD incubated with *M. lysodiekticus* cells; lane 4, untreated *Ts*CWBD.

For each sample, 8 µl 4× E-PAGE Loading Buffer (EPBUF-01, Life Technologies) and 1 µl (10×) NuPAGE Sample Reducing Agent (Life Technologies) were added to 2 µl supernatant. The two samples were then vortex mixed and heated in a heating block at 70°C for 10 min. 20 µl of each prepared sample was then loaded onto a Criterion XT 8–16% gradient Bis-Tris SDS–PAGE gel and run in Criterion XT MOPS buffer according to the manufacturer’s instructions (Bio-Rad). A Rainbow recombinant molecular-weight marker was also run in the gel (RPN800, GE Healthcare). The SDS–PAGE gel was stained with Simply Blue Coomassie stain (Life Technologies) and the results were visualized (Fig. 1 ▸).

### 2.6. Mutational study on *Ks*GH184

The activity of the E41A mutant of *Ks*GH184 was compared with that of wild-type *Ks*GH184 in an assay with fluorescein-labelled (FITC) *M. lysodeikticus* peptidoglycan (Maeda, 1980 ▸) at pH 6.0 and 30°C. Briefly, the assay measures lysozyme activity on *M. lysodeikticus* cell walls, which are labelled with fluorescein isothiocyanate (FITC) at the amino group of the peptide, resulting in the fluorescence being quenched. Lysozyme action can relieve this quenching, leading to a dramatic increase in fluorescence that is proportional to lysozyme activity. Supplementary Fig. S1 shows an increase of fluorescence for wild-type *Ks*GH184. In contrast to the wild-type *Ks*GH184, the E41A mutant had no activity on FITC-labelled peptidoglycan (Supplementary Fig. S1).

### 2.7. Crystallization and structure determination

For all protein samples, initial crystallization was carried out in a number of commercial screens using sitting-drop vapour diffusion with drops set up using a Mosquito Crystal liquid-handling robot (SPT Labtech, UK) with 150 nl protein solution plus 150 nl reservoir solution in 96-well format plates (MRC 2-well crystallization microplates, Swissci, Switzerland) equilibrated against 54 µl reservoir solution. All computations were carried out using programs from the *CCP*4 suite (Agirre *et al.*, 2023 ▸) unless otherwise stated. Data-collection and processing and final refinement statistics are given in Table 1 ▸. All structures were refined with *REFMAC* (Murshudov *et al.*, 2011 ▸) alternating with manual model correction in *Coot* (Emsley *et al.*, 2010 ▸). Structure figures were drawn with *CCP*4*mg* (McNicholas *et al.*, 2011 ▸). The quality of the final models was validated using *MolProbity* (Chen *et al.*, 2010 ▸).

#### 2.7.1. Full-length *Ts*CWBD-GH24

Several hits were obtained in the initial screens, mostly clusters. The best hit was condition C3 of the AmSO4 screen from Qiagen (0.2 *M* potassium fluoride, 2.2 *M* ammonium sulfate): a cluster of thick rods. These were separated as much as possible, cryoprotected with 3.3 *M* sodium malonate and tested in-house on a Rigaku MicroMax-007 X-ray generator (Cu *K*α, λ = 1.54179 Å) equipped with a MAR345 image-plate detector (MAR Research, Germany). Data were subsequently collected on beamline I04 at Diamond Light Source, processed using *XDS* (Kabsch, 2010 ▸) within the *xia*2 pipeline (Winter *et al.*, 2013 ▸) and scaled with *AIMLESS* (Evans & Murshudov, 2013 ▸).

A partial structure solution was obtained using the *BALBES* automated molecular-replacement (MR) pipeline (Long *et al.*, 2008 ▸), which generated a search model for the GH24 catalytic domain consisting of residues 138–225 from PDB entry 3hde (Sun *et al.*, 2009 ▸) and positioned two copies of this model. Because of the absence of MR models with sufficiently high sequence identity to CWBD, model extension involved density modification with *Parrot* (Cowtan, 2010 ▸) and model building with *Buccaneer* (Cowtan, 2006 ▸). Despite the significant spatial separation of the CWBD and GH24 domains belonging to the same polypeptide chain, the full-length dimers (these are actually two molecules in the asymmetric unit, with no evidence of them being a biological dimer) possess very accurate twofold symmetry that helped *Parrot* to extend the averaging mask from 32% to 46% of the asymmetric unit during iterative density modification that involved twofold averaging. The map quality was sufficient for *Buccaneer* to build the missing parts of the GH24 domains and almost complete CWBD domains (72% of residues in 11 fragments) in one go. *Coot* and *REFMAC*5 were used for subsequent iterative model correction and refinement. The final model statistics are shown in Table 1 ▸.

#### 2.7.2. The GH184 proteins and their SH3-like domains

The GH184 family was identified by the module-walking approach as described above. It should be noted that while the search was carried out for CBWD-linked new protein families, not all members of the newly identified families necessarily contained a CBWD, but sometimes could be standalone catalytic domains. One such protein without a CBWD was selected for initial crystallization experiments to facilitate crystal formation due to the absence of flexible interdomain linkers.

**Ks*GH184, a natural GH184 lacking a CWBD*. An initial hit was obtained in condition G10 of Crystal Screen 2 from Hampton Research (50 m*M* cadmium sulfate, 0.1 *M* HEPES pH 7.5, 1 *M* sodium acetate trihydrate). The conditions were optimized to give final crystals in 0.9 *M* sodium acetate, 0.1 *M* HEPES pH 7.5, 40 m*M* CdCl_2_. The crystals were cryoprotected by adding ethylene glycol mixed with mother liquor in a 1:2 ratio. Data were collected to 1.1 Å resolution on beamline I03 at Diamond Light Source and were processed using *XDS* (Kabsch, 2010 ▸) within the *xia*2 pipeline (Winter *et al.*, 2013 ▸) and scaled with *AIMLESS* (Evans & Murshudov, 2013 ▸). The structure was solved by SAD using the Cd atoms with the *Crank*2 pipeline (Pannu *et al.*, 2011 ▸).

*The catalytic domain of *Tt*GH184*. This time the goal was to crystallize the intact two-domain protein. Crystallization was carried out in the presence of 5 m*M* TCEP and crystals were obtained in condition D12 of the PACT screen [0.01 *M* zinc chloride, 0.1 *M* Tris pH 8, 20%(*w*/*v*) PEG 6000]. Ethylene glycol mixed with the well solution in a 1:2 ratio was used for cryoprotection (6 µl well solution + 3 µl ethylene glycol). Data were collected on beamline I04-1 at Diamond Light Source, processed using *XD*S (Kabsch, 2010 ▸) within the *xia*2 pipeline (Winter *et al.*, 2013 ▸) and scaled with *AIMLESS* (Evans & Murshudov, 2013 ▸). The structure was solved using *MOLREP* (Vagin & Teplyakov, 2010 ▸) using the natural catalytic GH184 domain from *Kionochaeta* sp. as the MR model. However, the structure corresponded to the GH184 domain alone, with the flexible linker presumably being cleaved during crystallization.

*The CWBD of *Pv*GH184*. As for the *T. terrestris* muramidase, the intention was to crystallize the intact two-domain protein. Initial minor hits were obtained in condition C7 of the JCSG screen [0.2 *M* zinc acetate dehydrate, 0.1 *M* sodium acetate, 10%(*w*/*v*) PEG 3000]. This crystalline material was used to prepare seeding stock, and microseed matrix screening (MMS; for a review, see D’Arcy *et al.*, 2014 ▸) was carried out using an Oryx robot (Douglas Instruments) according to published protocols (Shaw Stewart *et al.*, 2011 ▸; Shah *et al.*, 2005 ▸). Briefly, crystals were transferred onto a glass slide, crushed and collected in a Seed Bead (Hampton Research) with 50 µl well solution added, vortexed for 1 min and used as an initial seeding stock: unused seeding stocks were stored at −20°C for later experiments. MMS resulted in better formed but very small crystals in condition C9 of the PACT screen (0.2 *M* LiCl, 0.1 *M* HEPES pH 7.0, 20% PEG 6K). These crystals were tested in-house and diffracted to 4.5 Å resolution, but attempts to reproduce and optimize them were not successful, which caused a (correct) suspicion that the protein might have been cleaved by proteases during crystallization, which was impossible to test on a gel because of the very small number and small size of the crystals.

Data were collected on beamline I04 at Diamond Light Source. Automated data processing using the *xia*2 pipeline (Winter *et al.*, 2013 ▸) favoured space group *C*222_1_ but pointed to possible twinning. Not surprisingly, attempts at structure solution using the autoprocessed *C*222_1_ merged data failed. Therefore, the data were scaled and merged in space group *P*1 using *AIMLESS*, and an initial solution in *P*1 was obtained using *MOLREP* (Vagin & Teplyakov, 2010 ▸) with the CWBD of the GH24 CWBD muramidase from *T. saccata* as the search model. The correct *P*2_1_ symmetry was identified using *Zanuda* (Lebedev & Isupov, 2014 ▸) and the data were scaled and merged again using *AIMLESS* and the *P*2_1_ model from *Zanuda* as a reference structure. The *P*2_1_ model with two copies of the CWBD in the asymmetric unit was iteratively refined using *REFMAC*5 with the twin option switched on and was corrected using *Coot*. Inspection of the molecular packing using *Coot* showed that this pseudo-orthorhombic structure was an order–disorder structure (Dornberger-Schiff & Grell-Niemann, 1961 ▸), as illustrated in Supplementary Fig. S2, and indicated that the crystal was an order–disorder twin. Such twinning frequently presents additional complications for data processing and refinement owing to the small sizes of the twin domains. Diffraction images were visually inspected and some images revealed streaky spots that are characteristic of partially disordered crystals (another term for twins with small sizes of the twin domains). In addition, there were non-origin peaks in the Patterson maps at 0.12(**a** – **c**) consistent with the model of the twin interface in Supplementary Fig. S2. These observations are consistent with rather noisy solvent regions. However, the effect of the partial disorder was minor when compared with other cases (see, for example, Ponnusamy *et al.*, 2014 ▸), with the height of the non-origin Patterson peaks being only 6% compared with the origin peaks, and therefore data correction was not carried out.

#### 2.7.3. Triglycine complex of the GH24 family *Ts*CWBD

The aim here was to gain information on substrate binding by the SH3-like domains of GH24 and GH184 muramidases. Initially, co-crystallization with pentaglycine was tried, similar to the approach used for lysostaphin (PDB entry 5leo), but the peptide had very low solubility and could only be solubilized in citric acid pH 2.0, making a 20 m*M* solution, and the crystals did not contain the ligand. Therefore, the more soluble triglycine was tried as a ligand. Triglycine was dissolved in water and a 200 m*M* stock solution was made and added to the protein to a final concentration of 10 m*M*. Crystals were obtained in condition D10 of the MORPHEUS screen {0.12 *M* alcohols [1,6-hexanediol, 1-butanol, 1,2-propanediol (racemic), 2-propanol, 1,4-butanediol, 1,3-propanediol], buffer system 3 [Tris (base), Bicine, 30% EDO_P8K]} using MMS from Crystal Screen 2 condition C7/H7 [0.2 *M* ammonium sulfate, 30% PEG 4K, 0.2 *M* ammonium phosphate monobasic, 50%(*v*/*v*) (±)-2-methyl-2,4-pentanediol, Tris–HCl pH 8.5]. Data were collected on beamline I03 at Diamond Light Source, processed using *XDS* (Kabsch, 2010 ▸) and scaled with *AIMLESS* (Evans & Murshudov, 2013 ▸) as incorporated in *autoPROC* (Vonrhein *et al.*, 2011 ▸). The structure was solved using *MOLREP* (Vagin & Teplyakov, 2010 ▸) using the CWBD from *P. virgatum* as a search model.

### 2.8. Modelling

#### 2.8.1. Linker modelling for *T. saccata* muramidase

We used the *RosettaRemodel* application (Huang *et al.*, 2011 ▸) to model the missing linkers which connect the CWBD and GH24 domains of *T. saccata* muramidase in the asymmetric unit. For this, we defined a blueprint file that specifies all residues in the input structure as fixed, except for the loop start and end residues, and defines the missing linker residues for insertion between the loop start and end residues. Based on this blueprint file, the *RosettaRemodel* application performs fragment insertion from the *Rosetta* fragment database derived from the PDB (Berman *et al.*, 2000 ▸) to build the missing loop between the CWBD and GH24 domains in both chains of the asymmetric unit. The loop-building step is then followed by cyclic coordinate descent minimization to close the loop. This protocol was run for both options of pairing the CWBD and GH24 domains in the asymmetric unit from the crystal structure, with 1000 independent linker modelling trajectories each. The lowest energy model from these trajectories was then used as the representative model for the given domain-pairing option.

#### 2.8.2. Modelling of the intact full-length *T. terrestris* CWBD-GH184 molecule

To model the complete CWBD-GH184 molecule from *T. terrestris*, we used all five network models from *AlphaFold*2 that were created for CASP14 and validated for structure-prediction quality (Jumper *et al.*, 2021 ▸). Those network models are known to produce slightly different structural models due to small differences in their network architectures and parameters [for details, see Supplementary Table 5 of Jumper *et al.* (2021 ▸), Models 1.1.1, 1.1.2, 1.2.1, 1.2.2 and 1.2.3, as well as the config.py file in the alphafold/model/directory of the program]. For comparison, we also used the *RosettaCM* application (Song *et al.*, 2013 ▸) with our experimental X-ray structures of the individual domains, GH184 from *T. terrestris* and CWBD from *P. virgatum*, as templates for homology modelling. Providing the full amino-acid sequence of the *T. terrestris* CWBD-GH184 molecule as a target, the *RosettaCM* application automatically models the missing linker residues using fragment insertion from the *Rosetta* fragment database derived from the PDB (Berman *et al.*, 2000 ▸), followed by cyclic coordinate descent minimization to close the loop. This protocol was run for 10 000 independent trajectories. The five structural models from *AlphaFold*2 (see Section 3), as well as the five lowest energy models from these, were then superposed onto their respective GH184 domains to compare the linker conformations and relative placements of the CWBD domain.

## 3. Results and discussion

### 3.1. A fungal GH24 muramidase with a CWBD from *T. saccata*

A broad bioinformatic screening for new muramidases from known GH families (GH22–GH25; Taylor *et al.*, 2019 ▸; Moroz *et al.*, 2021 ▸) led to the discovery of an enzyme from *T. saccata* with an extra domain attached to the catalytic GH24 domain. As described below, we demonstrate that this is an SH3-like cell-wall-binding domain (CWBD).

Three different constructs of *Ts*GH24 were cloned, expressed and purified (see Table 2 ▸) as well as eight other examples of GH24 muramidases with a CWBD (see Table 3 ▸). A similarity tree based on amino-acid sequence alignment of the nine GH24s with a CWBD is shown in Supplementary Fig. S3.

The effect of the CWBD on muramidase activity was studied by comparing the activity in the OD-drop assay for several constructs of *Ts*CWBD-GH24 (Table 2 ▸).

There is a clear decrease in activity when the CWBD is removed. Combining equal amounts of the individual GH24-CD and CWBD domains was also investigated, but this did not recover the activity.

Several GH24s with the CWBD were also tested for muramidase activity using the reducing-ends assay (Table 3 ▸).

### 3.2. Evidence for cell-wall binding by the *Ts*CWBD

To elucidate the binding properties of *Ts*CWBD the binding domain was mixed with Avicel (a cellulose polymer) and with *M. lysodiekticus* cells. After incubation the supernatants were analysed by SDS–PAGE (Fig. 1 ▸).

The *Ts*CWBD protein migrates at about 10 kDa, as expected (lane 4), and the band intensity of *Ts*CWBD is approximately equal in the supernatant from the Avicel and in the untreated sample (lanes 2 and 4, respectively), while a clear reduction in the *Ts*CWBD content in the supernatant was seen after incubation with *M. lysodiekticus* cells (lane 3). This indicates binding of *Ts*CWBD to the insoluble *M. lysodiekticus* cells. At the start of this work, the component of the *M. lysodiekticus* cells to which the *Ts*CWBD binds was not known.

### 3.3. Crystal structure determination of full-length *Ts*GH24 muramidase

**Figure 2:**
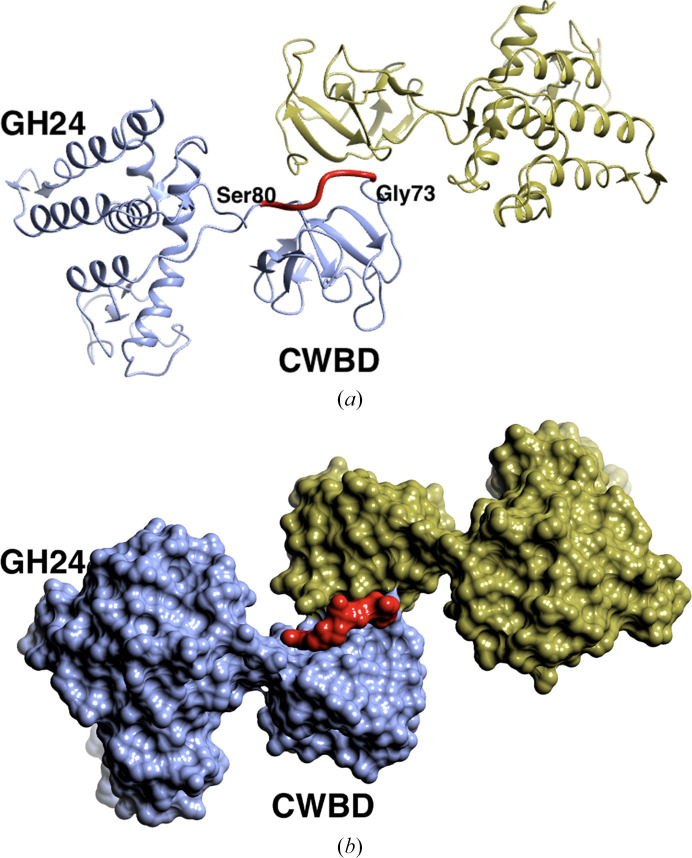
The two monomers of *T. saccata* muramidase in the asymmetric unit. (*a*) In ribbon format coloured by chain: chain *A*, ice blue; chain *B*, gold. There is insufficient electron density to define the position of the linker residues 74–79 between the GH24 and CWBD domains, which left some ambiguity as to which pairs form a monomer. (*b*) A surface plot of the experimental protein surface coloured by chain as in (*a*). The *RosettaRemodel* model of the linker for chain *A* is shown in red in both (*a*) and (*b*): note that this is a model not an experimental structure of the linker.

This is the first structure of a eukaryotic, fungal, GH24 muramidase. There are two independent monomers in the asymmetric unit corresponding to the expected full-length protein, each with two clearly identified domains: an N-terminal CWBD and a catalytic GH24 (Fig. 2 ▸ *a*). The linker, G73-SSSGGG-S80, appears to be flexible: its electron density is ill defined and it was not initially obvious how to assign the domains which compose a monomer. This was resolved by inspection of the surface, which strongly suggested a likely choice of connectivity for the domain pairs (Fig. 2 ▸ *b*). This was confirmed by computer modelling of the missing linkers in the asymmetric unit using the *RosettaRemodel* application (Huang *et al.*, 2011 ▸) as described in Section 2. For the GH24-CWBD domain-pairing option shown in Fig. 2 ▸, the lowest Rosetta energy of the linker from 1000 independent modelling trajectories was slightly lower (−10.825 REU versus −8.363 REU, respectively), indicating a more thermodynamically favourable conformation when the domains are connected as shown in the figure. For comparison, the image of the lowest energy model of the alternative domain-pairing option is shown in Supplementary Fig. S4. It does seem odd that the density is missing for the linker since in the model it is required to wrap rather tightly around the surface. One explanation could be that these residues have been cleaved during crystallization. While it is possible that the relative positions of the two domains is flexible in solution and that this particular orientation is a result of the crystal packing, we note that the linker is relatively short in this enzyme.

#### 3.3.1. The catalytic GH24 domain

Three structures of intact bacterial GH24 muramidases are currently present in the PDB, plus structures from six different bacteriophages, including the molecular-replacement model, endolysin R21 from phage 21 (Sun *et al.*, 2009 ▸), and a great number for T4 lysozyme. The overall fold of the *T. saccata* catalytic GH24 domain follows that of the homologous GH24 enzymes. A more detailed description of the catalytic domain and structure and sequence comparisons (Supplementary Figs. S5 and S6) with other family members is given in the supporting information.

#### 3.3.2. The SH3-like cell-wall-binding domain (CWBD)

**Figure 3:**
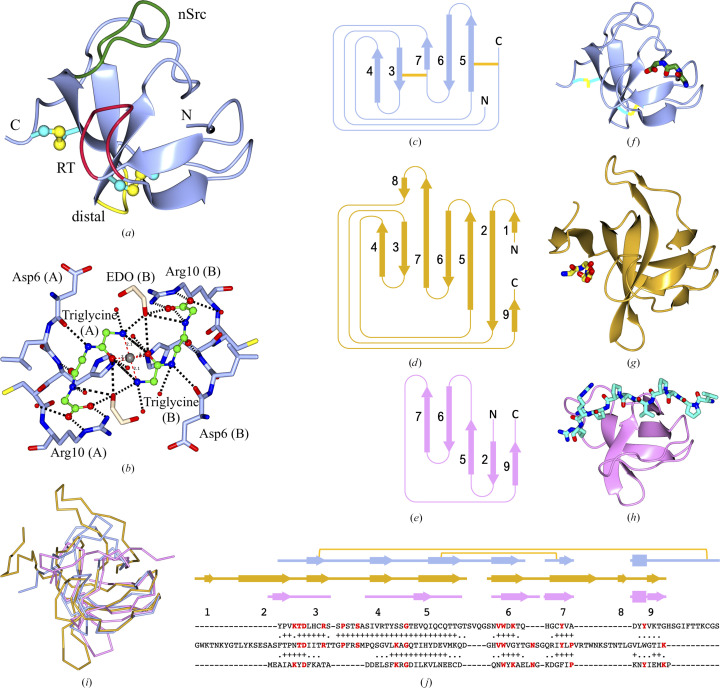
SH3 domain comparisons. (*a*) The overall fold of the *Ts*GH24 fungal muramidase SH3-like domain shown in ice blue. Loops potentially involved in target recognition are coloured red for the RT loop, green for the n-Src loop and yellow for the distal loop, which are named according to the standard SH3 convention. Two disulfide bridges, which are a specific feature of the SH3-like domains of fungal muramidases identified in the present study, are shown in ball-and-stick representation. (*b*) Peptide-binding site: triglycine forms salt bridges to the main-chain O atom of Asp6, the N atom of His8 and the side chain of Arg10. Two triglycine molecules (green) from the different subunits in the asymmetric unit coordinate a zinc ion together with two His8 residues from different chains, thus forming crystal contacts. In addition, there are two ethylene glycol molecules from the crystallization conditions that further stabilize the crystal contacts. The peptides are in ball-and-stick representation, the protein in ice blue and ethylene glycol molecules in light brown in cylinder representation. (*c*, *d*, *e*) Topology schemes and (*f*, *g*, *h*) complexes of SH3-like domains with (poly)peptides: (*c*, *f*) the *Ts*GH24 SH3-like domain in complex with triglycine, (*d*, *g*) the SH3b domain of lysostaphin from *Staphylococcus simulans* with pentaglycine (PDB entry 5leo, Table 2 ▸) and (*e*, *h*) the mouse Grb2 N-terminal domain with a decapeptide spanning both the canonical and bacterial binding sites (PDB entry 2gbq, Table 2 ▸). Proteins and ligands are shown as ribbons and cylinders, respectively. The long bending strand 2 in (*g*) is shown by two fragments of ribbon. (*i*) C^α^ traces of SH3-like domains (*f*, *g*, *h*) superposed using *GESAMT* (Krissinel, 2012 ▸) and (*j*) the corresponding sequence and secondary-structure alignments. Exact amino-acid matches are highlighted in red. The quality of 3D alignment is represented by symbols above the second and third amino-acid sequences. A plus sign means a C^α^ distance of less than 1.5 Å from the corresponding residue from the first sequence. A dot indicates that *GESAMT* treated the two residues as spatially aligned despite a greater distance. Strands in the secondary-structure alignment are numbered sequentially for the second structure and by correspondence in the first and the third structure. Colours and order are as in (*c*)–(*h*). The three-residue helical motifs following strand 8 are not labelled in (*j*) and are omitted in (*c*)–(*e*). The yellow lines in (*c*) and (*j*) show disulfide bonds.

The structure of this small 73-amino-acid domain is made up of a set of β-strands with associated loops (Fig. 3 ▸). Two disulfide bridges, Cys9–Cys53 and Cys33–Cys72 (Fig. 3 ▸ *a*, Supplementary Fig. S7), help to stabilize the structure, although they are most probably not essential for stability because they are absent in some of the homologous structures discussed below. However, they are conserved in all examples that we have identified of this CWBD. Structure comparisons using *GESAMT* (Krissinel, 2012 ▸) revealed a similarity to SH3 domains as mentioned in Section 1 (see Table 4 ▸ and Fig. 3 ▸).

Initially, SH3 and SH2 domains were described in the Src (Rous sarcoma virus) tyrosine kinase and were termed Src homology 2 (SH2) and Src homology 3 (SH3) because they were conserved in Src and Abl kinases; a fascinating historic description is given in Pawson (2004 ▸). In these kinases, SH1 is a catalytic domain and SH2 and SH3 are not required for catalytic activity but modulate protein activity and substrate recognition. Since their discovery, SH3 domains have been identified not only in intracellular proteins of eukaryotes but also in extracellular proteins, virus genes and prokaryotes.

SH3 domains have an open β-barrel fold, which consists of five to eight β-strands arranged as two tightly packed antiparallel β-sheets. The linker loop regions sometimes contain short helices and are responsible for recognition of the binding partners. They are termed the RT loop, n-Src loop and distal loop in the order of their occurrence between β-strands 1, 2, 3 and 4 (Fig. 3 ▸ *a*), which are historic names described in Noble *et al.* (1993 ▸) and references therein, where R and T are Arg and Thr residues proved to be important by mutations, ‘n’ is for ‘neuronal’ and distal is just the position of the third loop with respect to the conserved surface patch. The classical SH3 domain is usually found in proteins that interact with other proteins and it mediates the assembly of specific protein complexes, as reviewed in Dionne *et al.* (2021 ▸), Kurochkina & Guha (2013 ▸) and Feller (2001 ▸). In the fungal muramidases, the most likely function of these domains is cell-wall targeting, allowing the enzymes to recognize peptide fragments of target peptidoglycans. In prokaryotes, this function has been identified for the SH3-like (SH3b, bacterial) domains of the staphylococcal endopeptidases of *Staphylococcus capitis* (Lu *et al.*, 2006 ▸) and *S. simulans* (Mitkowski *et al.*, 2019 ▸), which cleave the cell walls of a number of competing staphylococci, including *S. aureus*. The SH3b domains of both enzymes specifically recognize pentaglycine cross-bridges, which are characteristic of most staphylococci. The lysostaphin native producer *S. simulans* expresses the Lif (lysostaphin immunity factor) protein, which incorporates the serine residues into the interpeptide bridges, protecting it from autolysis (Szweda *et al.*, 2012 ▸ and references therein). Mutational studies showed that lysostaphin retained its activity without the SH3b domain, but lost its ability to distinguish between *S. aureus* and *S. simulans* cells and to bind to the bacterial cell wall (Baba & Schneewind, 1996 ▸).

### 3.4. The *Ts*GH24 fungal muramidase SH3-like domain in complex with triglycine

To further investigate the function of the SH3-like domain, we tried binding triglycine as a potential mimic of a peptide bridge in peptidoglycan, by analogy with the pentaglycine shown to bind to lysostaphin (PDB entry 5leo), to probe for the location of the target binding site. The peptide-binding surface was initially suggested as a hydrophobic patch flanked by the n-Src and RT loops, based on structure analysis, where the SH3 domain in human Fyn (PDB entry 1shf) was compared with other structures known at the time (Noble *et al.*, 1993 ▸). Subsequently, ‘specificity pockets’ were identified for proline-rich peptides bound to the Src SH3 domain (Feng *et al.*, 1994 ▸; Lim *et al.*, 1994 ▸) and a canonical nomenclature for the binding sites was created (Yu *et al.*, 1994 ▸), with ligands termed class I and class II depending on the N–C direction of the peptide relative to the specificity pocket. Later, the term specificity pocket was expanded to specificity zone due to the increasing number of ‘atypical’ peptides bound in non­conventional locations to a growing number of diverse SH3 domains (reviewed in Saksela & Permi, 2012 ▸; Kurochkina & Guha, 2013 ▸). Structure comparisons between the *Ts*GH24 SH3-like domain with bound peptide and two other ligand-bound SH3 domains, one bacterial and one murine, from Table 4 ▸, are shown in Figs. 3 ▸(*c*)–3 ▸(*j*).

The peptide in the *Ts*GH24 fungal muramidase SH3-like domain is bound on a different face of the molecule to that in the lysostaphin complex, which is in agreement with the discussion in the lysostaphin study: the peptides in bacterial SH3b domains are found in a location remote from the canonical specificity zone of the eukaryotic proteins (Mitkowski *et al.*, 2019 ▸). The triglycine in our structure is bound within the canonical specificity zone (Figs. 3 ▸ *b* and 3 ▸ *f*–3 ▸ *h*). Two triglycines from two independent subunits in the asymmetric unit form contacts through zinc, which is unlikely to be biologically relevant, and was apparently present as a contaminant in the crystallization solutions or purification/storage buffer, although it was not an explicit component of the crystallization conditions (Fig. 3 ▸ *b*).

A thermal shift assay using differential scanning by the fluorimetry (nanoDSF) method was used to confirm that the interaction with triglycine is genuine rather than mediated by zinc ions (see the supporting information for details). The experiments were carried out with Chelex-treated protein and triglycine to make sure that there was no residual zinc in any solution, as well as for the untreated protein. The results show that at pH 8.5, which is the pH of the crystallization conditions, the thermal shift is present for both treated and untreated samples, implying ligand binding (Supplementary Fig. S8 and Table S2). Interestingly, the overall stability is unusually high for the untreated samples, possibly due to zinc binding, with treatment with Chelex bringing the *T*_m_ at pH 7.5 and 8.5 closer to ‘normal’ for the average protein (see the supporting information).

**Figure 4:**
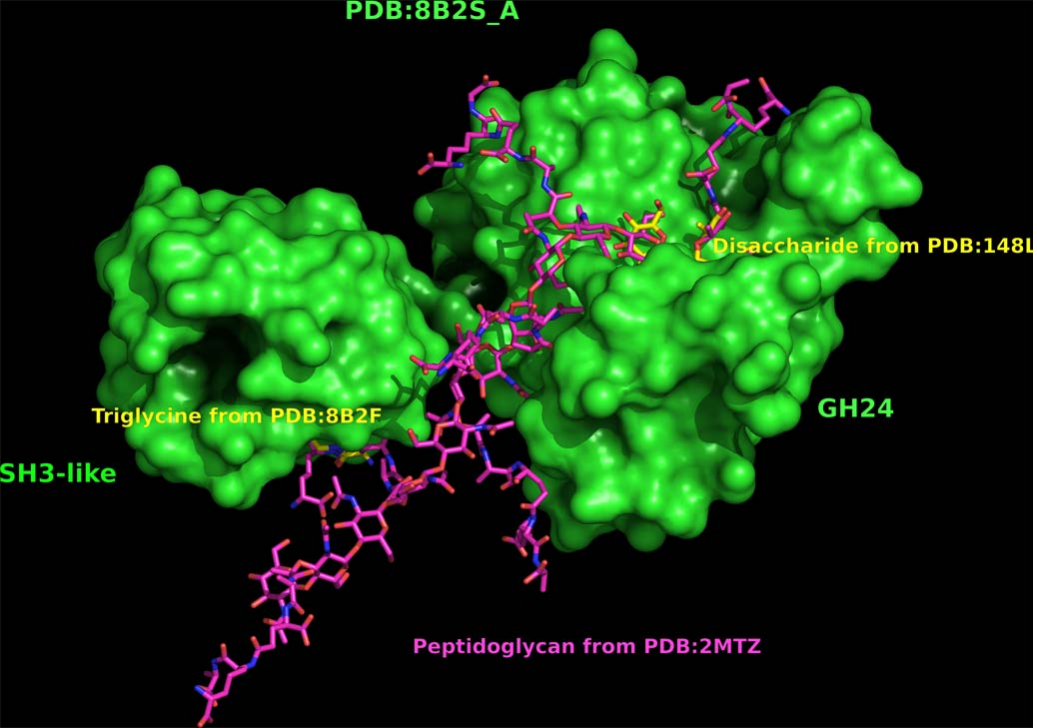
A manual model of peptidoglycan binding to the full-length *Ts*GH24 muramidase. Binding to peptidoglycan was guided by the disaccharide from PDB entry 148l (Kuroki *et al.*, 1993 ▸) and the triglycine from the present structure (PDB entry 8b2f). The protein is shown in surface representation in green and the ligands are shown as cylinders with peptidoglycan in purple and triglycine and disaccharide in cyan. This figure was generated in *PyMOL* (version 2.4.0; Schrödinger; https://www.pymol.org/).

Triglycine is just a model peptide, in contrast to the situation for lysostaphin, where pentaglycine is a known linker within the peptidoglycan of target organisms. However, the CWBD–triglycine structure demonstrates that a peptide ligand can be bound to this SH3 domain and it is located within the specificity zone. The result of manual docking of the peptidoglycan from PDB entry 2mtz (Schanda *et al.*, 2014 ▸), fitting the binding pockets of the GH24 and SH3-like domains, is shown in Fig. 4 ▸. This is of course just a hypothesis, but illustrates that the distances and geometries are about right for guiding the peptidoglycan molecule into the active site of the muramidase.

### 3.5. A new GH family of muramidases: GH184

#### 3.5.1. Module walking

**Figure 5:**
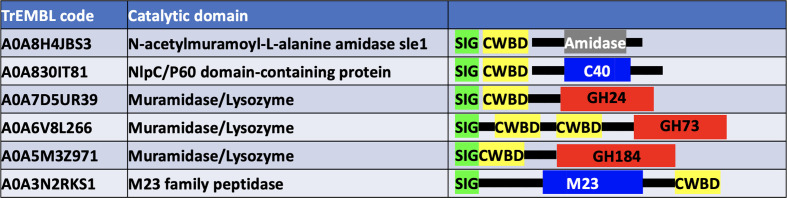
Schematic view of the domain structure of a representative set of enzymes containing a CWBD homologous to that found in *T. saccata* muramidase. These were identified by mining the TrEMBL database of sequences. Here, ‘Sig’ means signal peptide, amidase is an acylamide amidohydrolase, ‘C40’ is cysteine peptidase family 40, ‘M23’ is metallopeptidase family 23 and ‘GH24’ and ‘GH73’ are glycoside hydrolase families 24 and 73. All are concerned with the breakdown of bacterial cell-wall components. The TrEMBL code shown here for the GH184 family corresponds to the entry for a predicted SH3b domain-containing protein with the sequence derived from an EMBL/GenBank/DDBJ whole-genome shotgun (WGS) entry.

The ‘module-walking’ approach as described for LPMOs (Hemsworth *et al.*, 2014 ▸) was used to search sequence databases for other enzymes containing this SH3-like domain (Fig. 5 ▸).

The search resulted, *inter alia*, in the discovery of a potential new GH family of muramidases. We identified a significant number of fungal members of this family, and below we present three-dimensional structures of two separate catalytic domains and one SH3-like domain from three different fungal species. We now describe functional and structural studies of members of this family: the cloning and purification of the full-length protein from *T. terrestris* is described in the supporting information. It should be noted that bacterial family members also exist, but they lack a CWBD and are not discussed in the present study.

To expand the examples of GH184 members, a total of 15 muramidases were produced. Of these, 14 have a CWBD, while *Ks*GH184 is a natural enzyme without this domain (see Table 5 ▸). A similarity tree based on amino-acid sequence alignments of the 14 GH184 enzymes with a CWBD is shown in Supplementary Fig. S3(*b*).

#### 3.5.2. Evidence for muramidase activity

14 GH184s with the CWBD and one lacking this domain, *Ks*GH184, were tested for muramidase activity with a reducing-sugar assay (Table 5 ▸).

### 3.6. Crystal structures of the GH184 muramidases

#### 3.6.1. *Kionochaeta* sp. single catalytic domain GH184 muramidase

**Figure 6:**
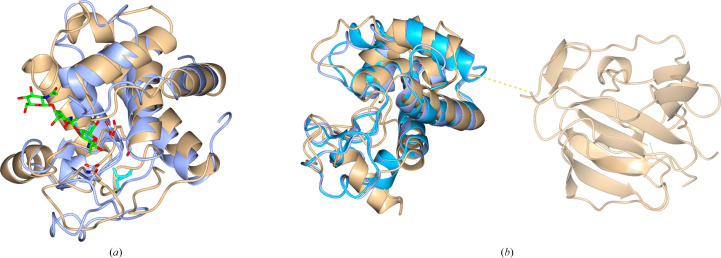
(*a*) Structure superposition of *Ks*GH184 (ice blue) and the cell-wall-degrading enzyme of the bacteriophage phi29 tail gp13 (PDB entry 3ct5, ligand complex, light brown). Glu41 (corresponding to the catalytic Glu45 in gp13) and Asp66 (superposed on the potentially ligand-stabilizing Gln54 in gp13) are shown as cylinders. Ethylene glycol molecules are shown as cylinders in cyan. (*b*) Superposition of both *Ks*GH184 and *Tt*GH184 on the catalytic domain of the full-length gp13; its cell-wall-binding domain differs from the SH3-like domain both in sequence and in structure (it was reported to be similar to LytM, a member of the peptidase M23 family; Firczuk *et al.*, 2005 ▸). The linker between the two domains of gp13 is poorly ordered, with residues 160–165 missing from the model, which reflects its flexibility, similar to the situation seen in the *Ts*GH24 enzyme (Fig. 2 ▸).

There is one subunit in the asymmetric unit, with two cadmiums, one with full occupancy, coordinated by His29 and His67 from the symmetry-related molecule and by four waters, and a second with an occupancy of 0.23, coordinated by His60 and five waters. There were no close sequence homologues with known X-ray structures for this enzyme. The closest structure, with a *GESAMT* *Q*-score of 0.36, is the N-terminal domain of a cell-wall-degrading enzyme in the bacteriophage phi29 tail, gp13 (PDB entries 3ct5 for the N-terminal, catalytic, domain and 3csq for the full length; Xiang *et al.*, 2008 ▸). Despite having a structural (and functional) similarity to GH184, this catalytic domain belongs to a different family from GH184; however, it has not yet been assigned a GH number in CAZy due to a lack of functional information (B. Henrissat, personal communication). Similar to that of gp13, the *Kionochaeta* GH184 domain is mostly an α-helix bundle (CATH; Sillitoe *et al.*, 2021 ▸). It can be roughly divided into two subdomains, with one subdomain all α-helical and the second, residues 42–88, containing two (or four in *Tt*GH184 described below) short β-strands and two very short α-helices with connecting loops. This subdomain differs more from gp13 (Figs. 6 ▸ *a* and 6 ▸ *b*) than the all-α subdomain, having only one, but a longer, helix and a different loop arrangement. This subdomain is most probably responsible for the substrate specificity. The substrate-binding pocket lies between the two subdomains and is indicated by ethylene glycol molecules in *Ks*GH184 and by NAG molecules bound in the ligand complex (PDB entry 3ct5). There are three poorly ordered ethylene glycol molecules from the cryoprotectant in the *Ks*GH184 structure, two of which are close to the active site. One of these ethylene glycol molecules occupies a similar location to one of the NAG molecules in PDB entry 3ct5 (Fig. 6 ▸ *a*).

A mutational study confirmed that Glu41 is essential for catalysis. Glu41 corresponds to the suggested catalytic Glu45 in gp13 and is located in the all-α subdomain at the end of helix 2 (Fig. 6 ▸ *a*). The less conserved aspartic acid, which is present in both hen egg-white (Asp52) and T4 lysozymes (Asp20), corresponds to Gly64 in gp13 (mentioned as Gly90 in the description in Xiang *et al.*, 2008 ▸; this is most probably a misprint) and to Ser69 in *Ks*GH184. The side chain of Asp66 is located close to that of Gln54 from gp13 (Fig. 6 ▸ *a*), which was suggested to be involved in stabilizing the substrate during catalysis (Xiang *et al.*, 2008 ▸).

#### 3.6.2. GH184 catalytic domain from *T. terrestris* muramidase

The full-length protein consists of an N-terminal CWBD followed by a catalytic domain. However, the CWBD was lost during crystallization, so the structure starts from Gly85 and only contains the catalytic domain. The fold is closely similar to that of *Ks*GH184, with the largest differences in the loop region close to the active-site entrance: the r.m.s.d. is 1.8 Å for 204–213 equivalent C^α^ positions in *Tt*GH184 (excluding residue 208), corresponding to residues 120–129 in *Kionochaeta* versus 0.75 Å for the full-length catalytic domains (superposed by *SSM* as incorporated in *Coot*; Krissinel & Henrick, 2004 ▸). There are two zinc ions from the crystallization medium, one coordinated by His132 (corresponding to the cadmium-coordinating His63 in *Ks*GH184), Glu135 and two waters, and the other coordinated by His170 from three symmetry-related molecules and possibly water, or some unidentified compound from crystallization/protein production. Glu125 in *Tt*GH184 corresponds to the catalytic Glu41 in *Ks*GH184 (Glu45 in gp13), and Asp150 in *Tt*GH184 (Asp66 in *Ks*GH184) corresponds to Gln54 in gp13 that potentially stabilizes the ligand.

### 3.7. The CWBD domain from the *P. virgatum* enzyme

Again, the aim was to crystallize the full-length enzyme, but as for the *T. terrestris* protein the domains were cleaved during the crystallization process. In contrast to *T. terrestris*, for this protein the crystals contained only the N-terminal CWBD. Its structure is similar to the CWBD from the *T. saccata* GH24 muramidase. There are two independent monomers in the asymmetric unit with a zinc ion bound between them; this zinc ion is a crystallization artefact. Two disulfide bridges are present in the same location as in *Ts*CWBD (Supplementary Fig. S7), which could add to the domain stability; however, they are not likely to be essential because the structurally similar SH3 domains lack these disulfide bridges. The second disulfide bridge could, however, play a role in target specificity because it brings the C-terminal loop into close proximity to the binding pocket (Supplementary Fig. S7). In addition, two ethylene glycol molecules are bound to each monomer.

### 3.10. Modelling of full-length CWBD-GH184 using *AlphaFold*2 and *RosettaCM*

**Figure 7:**
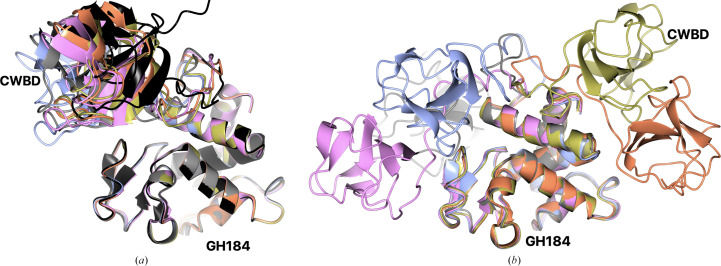
Modelling of full-length *Tt*CWBD-GH184. (*a*) Superposition of the top five *AlphaFold*2 models coloured ice blue (1), gold (2), coral (3), grey (4) and pink (5). The GH184 domain predictions superpose closely, with r.m.s.d.s of ∼0.5 Å. The CWBDs also superpose with one another with a similar r.m.s.d. and all in a similar position with respect to the GH184 domain. The *AlphaFold*2 model available from the AlphaFold2 database (A0A5M3Z971) for a protein from *A. terreus* superposed with the same set of models is shown in black. (*b*) Superposition of the top five *RosettaCM* models. Here, the relative orientation of the two domains differs widely in the five models, reflecting the long flexible linker. In both cases the superposition is based on the residues in the GH184 domain.

The *AlphaFold*2 (Jumper *et al.*, 2021 ▸) models of the complete CWBD-GH184 molecule from *T. terrestris* are quite similar in their relative domain placement and do not really show the full flexibility of the linker (Fig. 7 ▸ *a*). The *RosettaCM* (Song *et al.*, 2013 ▸) models with our solved structures of the individual domains, GH184 from *T. terrestris* and CWBD from *Penicillium virgatum*, showed a variety of possible CWBD domain orientations, supporting our hypothesis that the linker between the two domains is highly flexible (Fig. 7 ▸ *b*). It is likely that the linker adopts an extended conformation in solution and the models reflect the fact that both *AlphaFold*2 and *RosettaCM* tend to produce well packed models. The proposed flexibility of the linker explains the difficulty in obtaining crystals of the full-length protein and its apparent cleavage during crystallization experiments. The *P. virgatum* enzyme can be expected to have a similar extended and flexible linker.

In addition, an *AlphaFold*2 model is available from the AlphaFold2 database (A0A5M3Z971) for a protein from *Aspergillus terreus* annotated as an uncharacterized protein in the AlphaFold2 database and as an SH3b domain-containing protein in UniProt. One of its two domains aligns with the GH184 domains of the *Ks*GH184 and *Tt*GH184 X-ray structures (r.m.s.d.s of 0.82 and 0.67 Å), which are shown superposed with *AlphaFold*2 models of *Tt*CWBD-GH184 in Fig. 7 ▸, and the other aligns with the SH3-like domain in the X-ray structures of *Pv*GH184 and *Ts*GH184 (r.m.s.d.s of 0.54 and 0.69 Å, respectively; Supplementary Fig. S7). A flexible linker between the domains is in a compact conformation similar to the *AlphaFold*2 models of *Tt*GH184. This is most probably another member of the GH184 family.

## 4. Conclusions

Here, we have reported how a search for enzymes with muramidase activity for potential application as animal feed additives, which had previously led to the commercial product Balancius^TM^ for a GH25 enzyme from *Sodiomyces alcalophilus*, now led to the identification of a GH24 muramidase from the fungus *T. saccata*. Interestingly, the enzyme contained an additional N-terminal cell-wall-binding domain, which structure comparisons showed to have an SH3-like fold. The crystal structure of the intact enzyme was determined. Residues 74–79 of both protein chains in the asymmetric unit were disordered with no electron density, leading to some ambiguity in the connectivity between the two domains in each chain. This was resolved by inspection of the surface of the protein, which suggested the likely pairing of the domains, and the pairing was further confirmed by molecular modelling using *Rosetta Remodel* (Huang *et al.*, 2011 ▸). While it is not clear why there is no density for the linker in the crystal structure, this may suggest that the linker has been cleaved during crystallization: the relative orientation of the two domains may well be flexible in solution. This is the first structure of a fungal GH24 muramidase.

The use of the sequence of the *T. saccata* SH3-like CWBD in a ‘module-walking’ approach to search for homologous domains in other enzymes led to a significant number of novel hits. These were highly associated with activity on peptido­glycan, as seen in Fig. 5 ▸. One of these comprises a new family of glycoside hydrolases, which has now been assigned the number GH184 in the CAZy classification. Structural and functional studies were carried out on three fungal members of this family. The natural enzyme from *Kionochaeta* sp. lacks the CWBD and we describe its crystal structure. Bacterial members of family GH184 also lack a CWBD. Attempts were made to crystallize the full-length enzymes from *T. terrestris* and *P. virgatum*. However, in both cases the enzyme was cleaved at the much longer (than in the GH24 family) linker between the domains. The crystals of the *T. terrestris* enzyme contained only the catalytic GH184 domain, which was closely similar to that from *Kionochaeta* sp. The *P. virgatum* crystal, in contrast, only contained the SH3-like CWBD, which was similar in fold to the GH24 CWBD. *RosettaCM* modelling of the full-length GH184 CWBD molecule based on the experimental structures of the two domains suggested considerable flexibility in the extended linker.

The novel CBWDs of muramidases were first discovered through sequence analysis, but structure comparisons were essential to allow the conclusion that these domains belong to the SH3-like family. The structure of the complex with triglycine provided an additional argument in favour of these domains binding peptide bridges in peptidoglycan, similar to what was observed for the SH3b domains of lysostaphin, which *Staphylococcus capitis* (Lu *et al.*, 2006 ▸) and *S. simulans* (Mitkowski *et al.*, 2019 ▸) use in their competition with the other staphylococci. In the case of fungal muramidases, fungal species possibly do not compete, but rather feed on dead bacteria, still using the SH3-like domains to enhance binding to the cell walls.

To summarize, our work led to the identification of an SH3-like noncatalytic CWBD module in GH24 family muramidases, followed by the discovery of a new GH family using the module-walking approach. The same SH3-like CWBD was also found in a number of other peptidoglycan-active enzymes.

## Supplementary Material

Supporting information file. DOI: 10.1107/S2059798323005004/rr5233sup1.pdf

PDB reference: *Ks*GH184, 8b2e

PDB reference: *Ts*CWBD–triglycine complex, 8b2f

PDB reference: *Pv*CWBD, 8b2g

PDB reference: *Tt*GH184, 8b2h

PDB reference: *Ts*CWBD-GH24, 8b2s

## Figures and Tables

**Table 1:** Structure solution and refinement Values in parentheses are for the outer shell.

	*Ts*CWBD-GH24	*Ts*CWBD–triglycine	*Pv*CWBD†	*Ks*GH184	*Tt*GH184
Beamline	I04	I03	I04	I03	I04-1
Wavelength (Å)	0.979	0.976	1.282	0.976	0.916
Temperature (K)	100	100	100	100	100
Space group	*P*3_1_21	*P*1	*P*2_1_	*P*3_1_21	*P*6_3_
*a*, *b*, *c* (Å)	99.44, 99.44, 133.25	26.11, 27.234, 50.039	36.14, 59.82, 36.20	61.52, 61.52, 85.19	84.62, 84.62, 62.88
α, β, γ (°)	90, 90, 120	76.68, 81.27, 72.66	90, 112.13, 90	90, 90, 120	90, 90, 120
Total reflections	465913 (32193)	95959 (125)	84744 (2308)	670503 (15835)	132993 (14235)
Unique reflections	56987 (3797)	27938 (96)	21381 (731)	71381 (2466)	10656 (1106)
Completeness (%)	100.0 (100.0)	67.0 (4.7)	93.8 (66.8)	94.1 (66.4)	99.9 (99.7)
Multiplicity	8.2 (8.5)	3.4 (1.3)	4.0 (3.2)	9.4 (6.4)	12.5 (12.9)
*R*_meas_ ‡	0.083 (2.582)	0.029 (0.206)	0.108 (1.282)	0.049 (0.775)	0.133 (1.319)
*R*_p.i.m._ §	0.040 (1.232)	0.021 (0.145)	0.069 (0.835)	0.021 (0.369)	0.051 (0.505)
〈*I*/σ(*I*)〉	16.2 (0.9)	28.1 (3.0)	7.4 (1.0)	22.7 (2.1)	12.1 (1.6)
Resolution range¶ (Å)	46.6–1.94 (1.99–1.94)	48.5–1.18 (1.20–1.18) [1.49–1.45]	59.8–1.50 (1.53–1.50) [1.58–1.55]	53.3–1.10 (1.12–1.10) [1.18–1.16]	36.6–2.36 (2.45–2.36)
CC_1/2_ ††	0.999 (0.328)	0.999 (0.977)	0.995 (0.261)	0.999 (0.726)	0.999 (0.870)
Wilson *B* factor (Å^2^)	32.9	10.2	15.3	10.3	47.4
No. of reflections, working set	54075	26537	20307	67870	10153
No. of reflections, test set	2862	1391	1052	3492	492
Final *R*_cryst_	0.168	0.113	0.163	0.113	0.197
Final *R*_free_	0.201	0.146	0.173	0.124	0.221
Coordinate error‡‡ (Å)	0.107	0.047	0.015	0.019	0.206
No. of non-H atoms	4002	1322	1214	1281	1098
R.m.s.d.s					
Bond lengths (Å)	0.0104	0.0070	0.0125	0.0118	0.0073
Angles (°)	1.63	1.3960	1.90	1.6640	0.9720
Average *B* factors (Å^2^)					
Protein	Chain *A*, 46.59; chain *B*, 44.96	Chain *A*, 14.50; chain *B*, 13.90	Chain *A*, 19.03; chain *B*, 18.13	14.9	68.64
Ligand	—	Chain *H*, 15.243; chain *T*, 14.882	N/A	N/A	N/A
*MolProbity* score	0.87	0.65	0.52	0.78	0.73
Ramachandran plot					
Most favoured (%)	99.15	98.6	97.9	98.6	96.5
Allowed (%)	0.85	1.4	2.1	1.4	3.5
Outliers (%)	0.0	0.0	0.0	0.0	0.0
PDB code	8b2s	8b2f	8b2g	8b2e	8b2h

† The crystal was twinned; refinement was conducted using amplitude-based twin refinement in *REFMAC*.

‡ Diederichs & Karplus (1997 ▸).

§ Weiss *et al.* (1998 ▸).

¶ The outermost resolution shell with completeness >90% is shown in square brackets if this is not the absolute outermost shell.

†† Karplus & Diederichs (2012 ▸).

‡‡ *R*-factor-based coordinate DPI (equation 26 in Cruickshank, 1999 ▸).

**Table 2:** Muramidase activity (OD-drop) of *Ts*GH24 with and without the CWBD CWBD-GH24 was dosed at 6.8 µg ml^−1^ and GH24-CD was dosed at 4.5 µg ml^−1^ to compensate for the difference in molecular weight. The molecular weight of CWBD-GH24 is 26 kDa and that of GH24-CD is 18 kDa. CWBD was dosed at 4.5 µg ml^−1^. The OD-drop at pH 6.0 was measured after 60 min.

*Ts*GH24 construct	Amino-acid residues of NCBI ID ON783686	OD-drop
CWBD-GH24	1–245	0.50
GH24-CD	81–245	0.17
CWBD	1–73	−0.03

**Table 3:** Muramidase activity (reducing-ends assay) for GH24s with a CWBD

Organism	NCBI ID	OD_405_ (pH 5.0, 0.7 µg ml^−1^)	OD_405_ (pH 5.0, 5 µg ml^−1^)
*Trichophaea saccata* (*Ts*CWBD-GH24)	ON783686	2.76	3.23
*Thermochaetoides thermophila*	ON783687	2.84	4.34
*Trichoderma harzianum*	ON783688	1.79	2.93
*Trichophaea minuta*	ON783689	2.19	2.48
*Chaetomium* sp. ZY287	ON783690	1.00	2.50
*Mortierella* sp. ZY002	ON783691	4.11	5.57
*Metarhizium *sp. XZ2431	ON783692	3.66	4.80
*Geomyces auratus*	ON783693	0.43	1.55
*Ilyonectria rufa*	ON783694	0.88	2.40

**Table 4:** Structures closest to the cell-wall-binding domain identified using *GESAMT* The structures are sorted by *GESAMT* *Q*-score, with a 0.399 cutoff.

				Amino acids			
PDB code	*Q*-score	R.m.s.d. (Å)	Sequence identity	Aligned	Total	Name, species	Reference
2kyb	0.4727	1.6598	0.3269	52	60	CpR82G, *Clostridium perfringens*	—
2krs	0.4719	1.1383	0.2222	54	74	SH3 domain of CPF_0587, *C. perfringens*	—
2kt8	0.4712	1.0197	0.2963	54	76	CPE1231 (468–535)	—
2p4r	0.4109	1.7470	0.1277	47	55	SH3 domain, rat, complex with AIP4-derived peptide	Janz *et al.* (2007 ▸)
2gnc	0.4095	1.7586	0.1277	47	55	srGAP1 SH3 domain, mouse	Li *et al.* (2006 ▸)
4glm	0.4075	1.7131	0.1064	47	56	SH3 domain of DNMBP, human	—
2gbq	0.4068	1.9408	0.1224	49	57	Grb2 N-terminal SH3 domain, mouse, complex with ten-residue peptide	Wittekind *et al.* (1997 ▸)
2x3x	0.4051	1.7333	0.1277	47	56	Syndapin 1 SH3, mouse	Rao *et al.* (2010 ▸)
1w6x	0.4043	1.5880	0.1087	46	56	SH3 domain of p40phox, human	Massenet *et al.* (2005 ▸)
6b29	0.4014	1.5498	0.1087	46	57	Second SH3 domain of STAC3, human	Wong King Yuen *et al.* (2017 ▸)
1csk	0.4003	1.9365	0.2245	49	58	CskSH3, human	Borchert *et al.* (1994 ▸)
4z88	0.4000	1.6576	0.1020	49	63	SH3-II of Rim-binding protein, *Drosophila*	Siebert *et al.* (2015 ▸)
5leo	0.3998	1.4676	0.1550	58	93	Lysostaphin SH3b domain + pentaglycin, *Staphylococcus simulans*	Mitkowski *et al.* (2019 ▸)

**Table 5:** Muramidase activity (reducing-sugar assay at two concentrations) of a number of GH184s

Organism	NCBI ID	OD_405_ (pH 5.0, 0.7 µg ml^−1^)	OD_405_ (pH 5.0, 0.7 µg ml^−1^)
*Penicillium simplicissimum*	ON783672	2.4	5.4
*Penicillium vasconiae*	ON783673	2.0	4.4
*Talaromyces proteolyticus*	ON783674	2.7	5.2
*Aspergillus* sp. XZ2668	ON783675	1.4	2.4
*Penicillium antarcticum*	ON783676	3.2	6.7
*Penicillium wellingtonense*	ON783677	2.2	3.9
*Penicillium roseopurpureum*	ON783678	1.8	3.1
*Penicillium virgatum* (*Pv*GH184)	ON783679	4.6	7.8
*Aspergillus niveus*	ON783680	6.0	8.7
*Chaetomium* sp. ZY369	ON783681	5.7	8.6
*Talaromyces atricola*	ON783682	2.9	5.4
*Trichocladium asperum*	ON783683	4.8	7.8
*Keithomyces carneus*	ON783684	3.1	5.1
*Thermothielavioides terrestris* (*Tt*GH184)	ON783685	3.9	8.5
*Kionochaeta* sp. (*Ks*GH184)	ON808694	0.05	0.45
