# Supplementary material for: Module walking using an SH3-like cell-wall-binding domain leads to a new GH184 family of muramidases
Source: Acta Crystallogr D Struct Biol. 2023 Jul 10;79(Pt 8):706–20. doi: 10.1107/S2059798323005004 (PMC10394673; doi:10.1107/S2059798323005004)
Supplement: Supplementary file 1 [file d-79-00706-sup1.pdf]

STRUCTURAL
BIOLOGY

Volume 79 (2023)

Supporting information for article:

Module walking using an SH3-like cell-wall-binding domain leads to a new GH184 family of muramidases

Olga V. Moroz, Elena Blagova, Andrey A. Lebedev, Lars K. Skov, Roland A. Pache, Kirk M. Schnorr, Lars Kiemer, Esben P. Friis, Søren Nymand-Grarup, Li Ming, Liu Ye, Mikkel Klausen, Mariianne T. Cohn, Esben G. W. Schmidt, Gideon J. Davies and Keith S. Wilson

S1. Methods

S1.1. Cloning, expression and purification of GH24 and GH184 muramidases

New GH24/GH184 muramidases with a CWBD were cloned and expressed by established protocols. Details about GH24 muramidases are described in (Liu Y., 2017), while GH184 muramidases are described in Liu *et al.*, 2018 (Liu, 2018). The most important features of the muramidase production are listed in Table S1 which also includes the NCBI ID codes. As examples of the procedures, details about the cloning, expression and the purification of *Ts*CWBD-GH24 and *Tt*GH184 are given in the following sections. The relationship (based on amino acid sequence) between the different new muramidases (GH24 and GH184, respectively) is illustrated in Figures S3a and S3b.

S1.2. Cloning and expression of *Ts*CWBD-GH24 and *Tt*GH184

*Ts*CWBD-GH24 and *Tt*GH184 were cloned and expressed in *Aspergillus oryzae* using genomic DNA. The genomic DNA was isolated according to standard procedures (WO2018113745) and the raw reads of were assembled using the program Idba (Peng *et al.*, 2010). The assembled sequences were analysed using standard bioinformatics methods for gene identification and function prediction. GeneMark-ES fungal version (Ter-Hovhannisyan *et al.*, 2008) was used for gene prediction. Blastall version 2.2.10 (Altschul *et al.*, 1990) (ftp://ftp.ncbi.nlm.nih.gov/blast/executables/release/2.2.10/) and HMMER version 2.1.1 (National Center for Biotechnology Information (NCBI), Bethesda, MD, USA) were used to predict function based on structural homology.

S1.2.1. *Ts*CWBD-GH24

The entire open reading frame encoding the *Ts*CWBD-GH24 gene was amplified from genomic DNA of strain *Trichophaea saccata* CBS804.70 using the following primer pair:
F-80470 5'- ACACAACTGGGGATCCACCATGCACGCTCTCACCCTTCT -3'
R-80470 5'- CTAGATCTCGAGAAGCTTTTAGCACTTGGGAGGGTGGG -3'
The fragments were then cloned into BamHI and HindIII digested pDau109 (WO2005042735) using an IN-FUSION™ Cloning Kit. Protoplasts of *A. oryzae* MT3568 were prepared and used for transformation of the plasmid according to WO 95/002043. Transformants were spore-purified twice before fermentation in shake flasks. Analysis of the culture fluid from the fermentations by SDS–PAGE confirmed the presence of a protein band migrating at the expected rate for the molecular size (22 kDa).

S1.2.2. *Tt*GH184

Strain *Thermothielavioides terrestris* NRRL 8126 was purchased from ATCC and PCR performed from Genomic DNA with the following primer pair:
KKSC0972F- 5’-CTATATACACAACTGGGGATCCACCATGCAGCTCTCCCTCCTCGT-3’

KKSC0972-R- TAGAGTCGACCCAGCCGCGCCGGCCATTACAACCCACCAGCCTGGC-3’
The fragments were then cloned into BamHI-HindIII digested pDau109, transformed, selected and culture fluid was analyzed as for *Ts*CWBD-GH24 (see above). The presence of a 25 kDa protein band on an SDS-PAGE confirmed the presence of the protein.

S1.3. Purification of *Ts*CWBD-GH24 and *Tt*GH184

S1.3.1. *Ts*CWBD-GH24

The fermentation supernatant was filtered through a Fast PES 20 bottle top filter with a 0.22 µm cut-off. The resulting solution was diafiltrated with 5 mM Na-acetate, pH 4.5 and concentrated (volume reduced by a factor of 10) on an Ultra Filtration Unit (Sartorius) with a 10 kDa cut-off membrane. Following this pre-treatment approximately 275 ml of the muramidase-containing solution were purified by cation-exchange chromatography on SP Sepharose (approximately 50 ml) in a XK26 column, using 50 mM Na-acetate pH 4.5 as buffer A, and 50 mM Na-acetate plus 1 M NaCl pH 4.5 as buffer B. The elution was performed with a 0-100% gradient of buffer B over 10 column volumes. Fractions were pooled based on the chromatogram (absorption at 280 and 254 nm) and SDS-PAGE analysis.
The molecular weight, as estimated from SDS-PAGE, was approximately 27 kDa and the purity was > 90%. Intact molecule mass spectrometry confirmed the expected amino acid sequence (calculated: 26205.5 g/mol, observed: 26205.3 g/mol).

S1.3.2. *Tt*GH184

The fermentation supernatant was filtered through a Fast PES 20 bottle top filter with a 0.22 µm cut-off. A 250 ml filtered fermentation sample was diluted with 250 ml MilliQ water and pH was adjusted to 4.5. The muramidase-containing solution was purified by cation-exchange chromatography on Capto S (approximately 30 ml) in a XK16 column, using as buffer A 50 mM Na-acetate, pH 4.5, and as buffer B 50 mM Na-acetate + 2 M NaCl, pH 4.5. The elution was performed with a 0-100% gradient of buffer B over 10 column volumes. Fractions were pooled based on the chromatogram (absorption at 280 and 254 nm) and SDS-PAGE analysis.
The molecular weight, as estimated from SDS-PAGE, was approximately 25 kDa and the purity was > 90%. Intact molecule mass spectrometry confirmed the expected amino acid sequence (calculated: 23823.5 g/mol, observed: 23824.9 g/mol).
Genbank entries for the wild type proteins studied here can be found in the Table S1. An E41A mutant of *Ks*GH184 was produced and purified with the same methods used for *Ks*GH184

S1.4. Thermal shift assay by nanoDSF for *Ts*CWBD tryglycine binding

The protein was diluted to 1mg/ml in buffers with three different pHs: 20 mM Na-acetate pH 4.5, 100 mM NaCl; 20 mM Tris-HCl pH 7.5, 100 mM NaCl; 20 mM Tris-HCl pH 8.5, 100 mM NaCl.

Triglycine (Sigma G1377) was diluted in water to make a 100 mM stock solution. The measurements were made using the Prometheus nanoDSF instrument (NanoTemper Technologies, Munich, Germany), with 10 µl samples in capillaries. The final experiment for Chelex 100 (Sigma) treated samples was run in duplicates, with single runs for the initial Chelex-treated, and the untreated samples. The results are shown in Table S2, and Fig. S8.

S2. Results

S2.1. The catalytic GH24 domain: comparisons with other family members.

The catalytic domain can be split into two subdomains Fig. S5 (a) and (b). There is a short helix after the N-terminus followed by a subdomain composed of mainly extended chains seen on the right. This is linked by a long helix to the left-hand subdomain in the Figure which is composed of α-helices. The position of the active site can be expected to be the large cleft between these domains which is confirmed by the binding site for the substrate peptidoglycan fragment (NAM-NAG)-LAla-DGlu-DAP-DAla) seen in a complex of T4 lysozyme, 148L (Kuroki *et al.*, 1993), shown in the same orientation, Fig. S5(c) and (d). The cleft in *Ts*GH24 is considerably wider than in the T4 structure (which is similar for both apo and ligand-bound forms, so this is not due to the absence of the ligand). We note that the peptidoglycan-bound T4 is the only GH24 structure with a complex of the enzyme with a ligand, the difficulty in synthesising such ligands doubtless being the reason for the lack of complexes for these enzymes. The sequences of some representative GH24 structures are shown in Fig. S6. The identity of the catalytic residues was defined for the representative T4 lysozyme (Kuroki *et al.*, 1999): this paper also describes how a mutation changes the enzyme from its natural inverting mechanism into a retaining one.
Superposition of the *Trichophaea saccata* structure on those of the three bacterial GH24 muramidases currently in the PDB (6ET6, 6H9D, 4ZPU) and four representative bacteriophage (2ANX, 3HDE, 7M5I, 1XJT) reveals rms differences of 1.6-2.0Å for 125-138 equivalent Cα atoms. A subset of these is superimposed in Fig. S5(b) with sequence alignment for a bigger subset shown in Fig. S6. The α-helical domain can be seen to be highly conserved in structure, while there are significant variations in the β-strand/extended chain subdomain conformation on the right, which results in considerable differences in the size of the substrate binding cleft. Superposition on the T4 lysozyme (PDB 148L), shows a somewhat larger rms of 3.0Å over 122 Cα atoms. All these structures in the PDB have only a GH24 domain. The catalytic glutamate is seen to lie in the same position in the structures shown in Fig. S5(b). In contrast, in the *T. saccata* structure the proposed catalytic Asp107 lies too far away from the Glu98. In the other structures the two residues are separated by about 9Å, the expected spacing for an inverting mechanism for a GH. It appears that the crystals contain an open form of the GH24, with the enzyme expected to close up to enclose the substrate. This is in keeping with the substantial variation in conformation of the right-hand domain in Fig. S5(b).

Table S1 . Muramidases (organisms, NCBI accession codes and production details (vector and purification method))

**Organism**	**NCBI ID**	**Vector**	**Purification method**
**GH24**			
Trichophaea saccata=TsCWBD-GH24	ON783686	pDau109	Cation exchange
Thermochaetoides thermophila	ON783687	pCaHj505	Cation exchange
Trichoderma harzianum	ON783688	pDau109	Cation exchange
Trichophaea minuta	ON783689	pCaHj505	Cation exchange
Chaetomium sp. ZY287	ON783690	pDau109	Cation exchange
Mortierella sp. ZY002	ON783691	pCaHj505	Cation exchange
Metarhizium sp. XZ2431	ON783692	pCaHj505	Cation exchange
Geomyces auratus	ON783693	pCaHj505	Cation exchange
Ilyonectria rufa	ON783694	pCaHj505	Cation exchange
**GH184**			
Penicillium simplicissimum	ON783672	pCaHj505	Cation exchange
Penicillium vasconiae	ON783673	pCaHj505	Cation exchange
Talaromyces proteolyticus	ON783674	pCaHj505	Anion exchange
Aspergillus sp. XZ2668	ON783675	pCaHj505	Anion exchange
Penicillium antarcticum	ON783676	pCaHj505	Cation exchange
Penicillium wellingtonense	ON783677	pCaHj505	Anion exchange
Penicillium roseopurpureum	ON783678	pCaHj505	Anion exchange
Penicillium virgatum=PvGH184	ON783679	pCaHj505	Cation exchange
Aspergillus niveus	ON783680	pCaHj505	Cation exchange
Chaetomium sp. ZY369	ON783681	pCaHj505	Cation exchange
Talaromyces atricola	ON783682	pCaHj505	Anion exchange
Trichocladium asperum	ON783683	pCaHj505	Cation exchange
Keithomyces carneus	ON783684	pCaHj505	Cation exchange
Thermothielavioides terrestris=TtGH184	ON783685	DORA	Cation exchange

Vector pDau109 is described in WO2005042735

Vector pCaHj505 is described in WO2013029496

DORA= Direct Overlap Recombination in *Aspergillus* (WO2018206001)

Table S2 Thermal shift assay by nanoDSF for *Ts*CWBD – triglycine binding

Samples non-treated, single runs.

pH[a]	Triglycine (mM)	T onset °C	Tm °C
4.5	0	75.2	88.2
	1	75.4	88.5
	10	76.2	88.9
	20	76.4	89.2
8.5	0	67.6	81.2
	1[b]	-	-
	10	73.5	86.1
	20	75.0	87.1

Samples Chelex-treated, run in duplicates.

pH	Triglycine (mM)	T onset °C	Tm °C
4.5	0	73.8 ± 0.1	85.25 ± 0.15
	1	73.6 ± 0.1	85.1 ± 0
	10	72.65 ± 0.05	84.55 ± 0.05
	20	72.6 ± 0.3	84.5 ± 0.2
8.5	0	54.25 ± 0.05	71.3 ± 0
	1	54.2 ± 0.2	71.85 ± 0.05
	10	61.4 ± 0.1	75.45 ± 0.05
	20	63.45 ± 0.35	76.95 ± 0.05

[a] Only the results for pH 8.5 and 4.5 are shown, the numbers for pH 7.5 were very similar to those for 8.5.

[b] Result not shown – experimental error for this sample

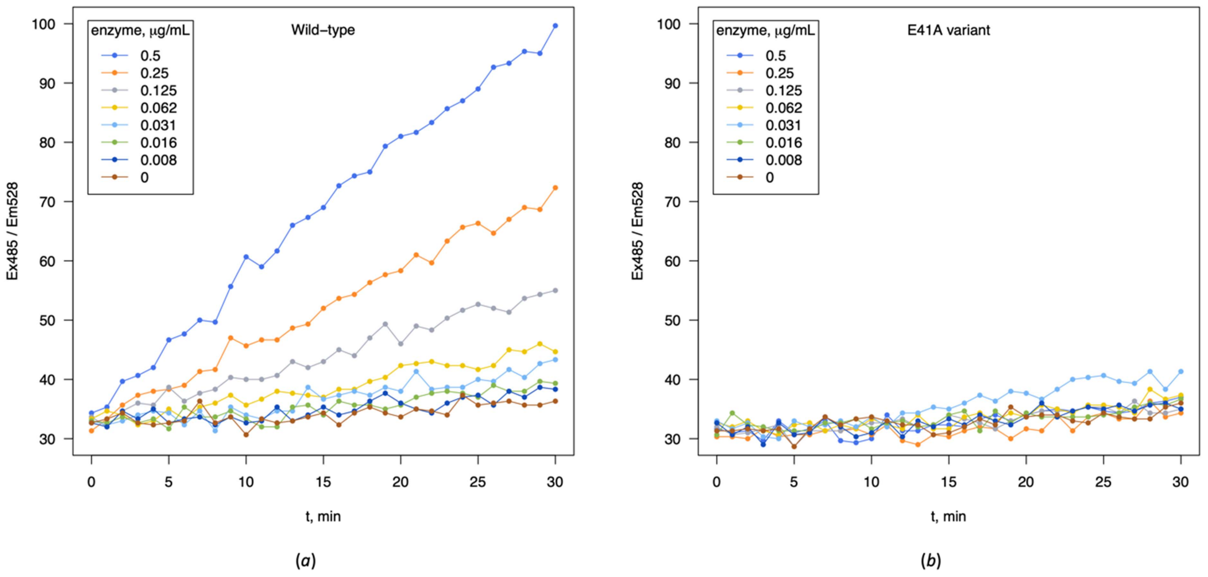

Figure S1 Loss of lysozyme activity for the E41A mutant of *Ks*GH184. The activities of (a) wild-type and (b) E41A variants of *Ks*GH184 were measured by the assay with *M. lysodeikticus* peptidoglycans labelled with fluorescein isothiocyanate. Increasing amounts of the enzyme were incubated with peptidoglycan labelled with fluorescein isothiocyanate for 30 minutes at 30C. The fluorescence was measured in a fluorescence microplate reader using excitation/ emission of ~485/530 nm.

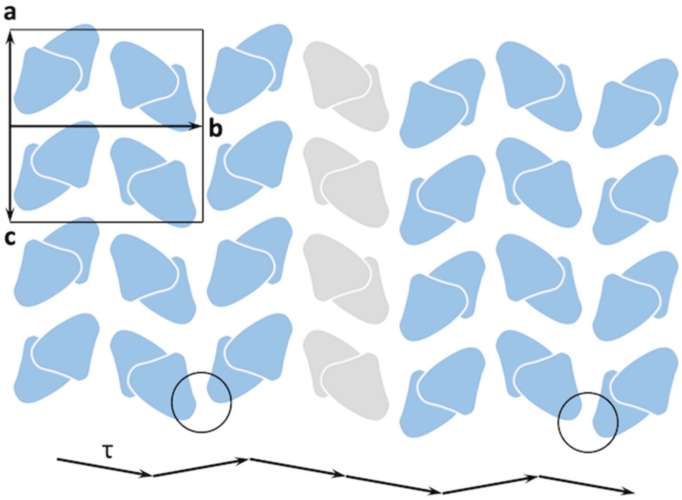

Figure S2 Putative structure of the twinned crystal of *Pv*CWBD. The scheme shows two fragments of $P2_1$ twin domains (blue) separated by an OD-layer forming the twin interface (grey). Columns of molecules form OD-layers, with stacking vectors shown at the bottom, and the circles highlight the asymmetry of the intermolecular contacts responsible for packing ambiguity and packing defects causing twinning. The arrangement shown is consistent with the non-origin Patterson peaks at 0.12 (**a** – **c**). The groupoid symmetry of the entire crystal can be described in the *C*-centred cell (base vectors **a**+**c**, **a**–**c** and **b**) by the symbol $C21(1)$ / $\{12_{0.12}(2_2)\}$ (Dornberger-Schiff & Grell-Niemann, 1961) which means that the OD layers have internal symmetry $C21(1)$ and the crystal would have $C222_1$ symmetry if the OD layers had been displaced by 0.06 **b** of the *C*-centred cell.

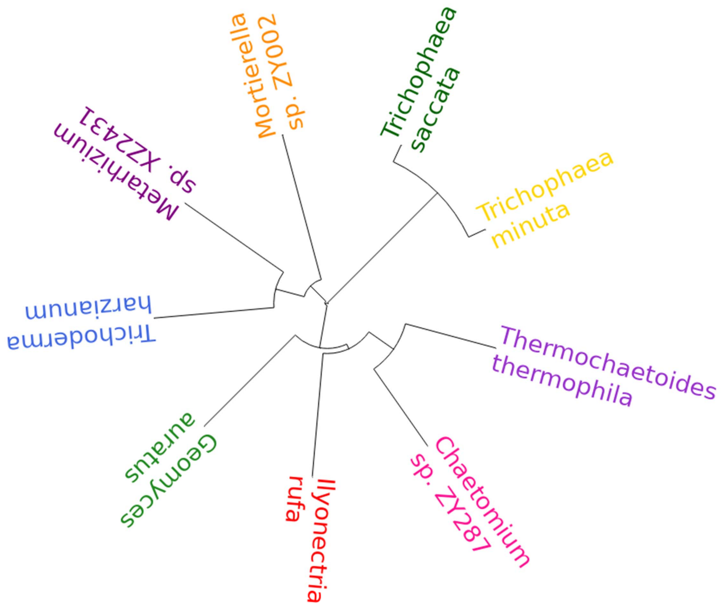
Mortierella sp. ZY002
Trichophaea saccata
Metarhizium sp. XZ2431
Trichophaea minuta
Trichoderma harzianum
Thermochaetoides thermophila
Geomyces auratus
Ilyonectria rufa
Chaetomium sp. ZY287

(a)

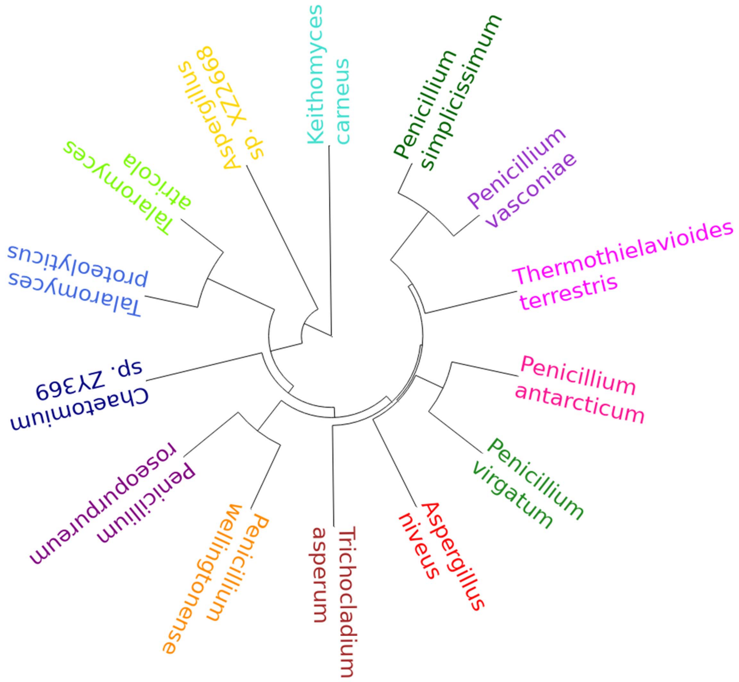
Keithomyces carneus
Aspergillus sp. XZ2668
Penicillium simplicissimum
Talaromyces atricola
Penicillium vasconiae
Talaromyces proteolyticus
Thermothielavioides terrestris
Chaetomium sp. ZY369
Penicillium antarcticum
Penicillium roseopurpureum
Penicillium virgatum
Penicillium wellingtonense
Trichocladium asperum
Aspergillus niveus

(b)

Figure S3 **(a).** A relationship tree based on amino acid sequence alignment of 9 GH24s with a CWBD. **(b).** A relationship tree based on amino acid sequence alignments of 14 GH184 enzymes**.**

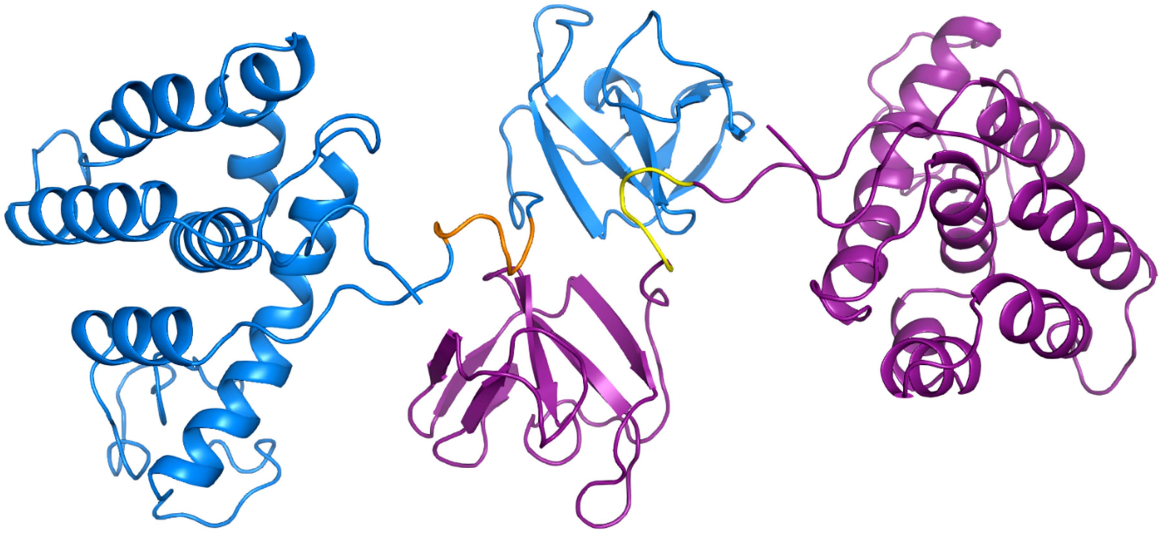

Figure S4 A domain-pairing option for *Ts*GH24 muramidase modelled using the RosettaRemodel application, alternative to the chosen model presented in the main text (Fig. 2). One chain of the dimer is in blue, with the linker shown in orange; another chain is in purple, linker is in yellow, the molecules are in ribbon representation.

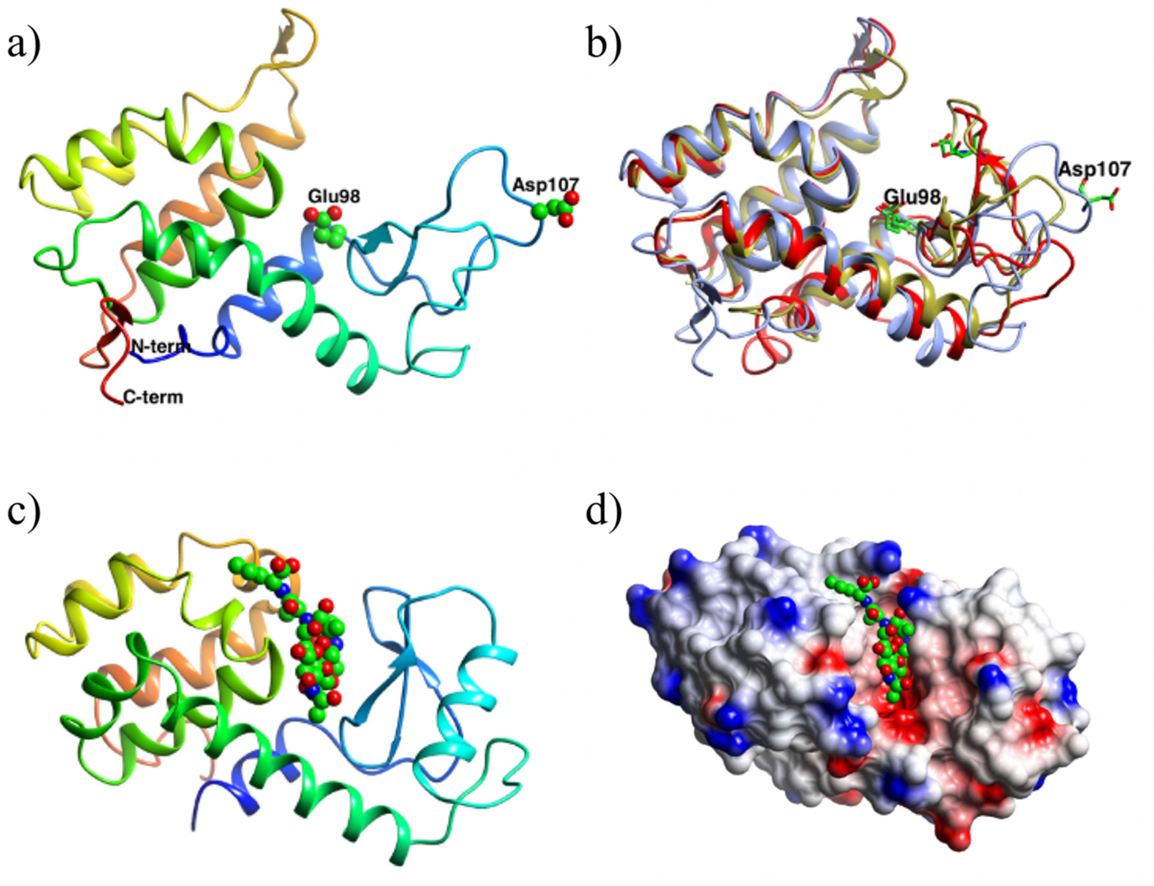

Figure S5 GH24 Domains. (a) Structure of the *Trichophaea saccata* GH24 catalytic domain. (b) The structure shown in ice blue is superimposed with two homologous GH24 enzymes from *Acinetobacter baumannii* (6ET6), in gold and Enterobacteria phage P21 (3HDE) in red. (c) The structure in the same orientation as the T4 lysozyme with a T26E mutation in complex with a substrate analogue (148L). (d) as (c), with the surface of the protein shown.

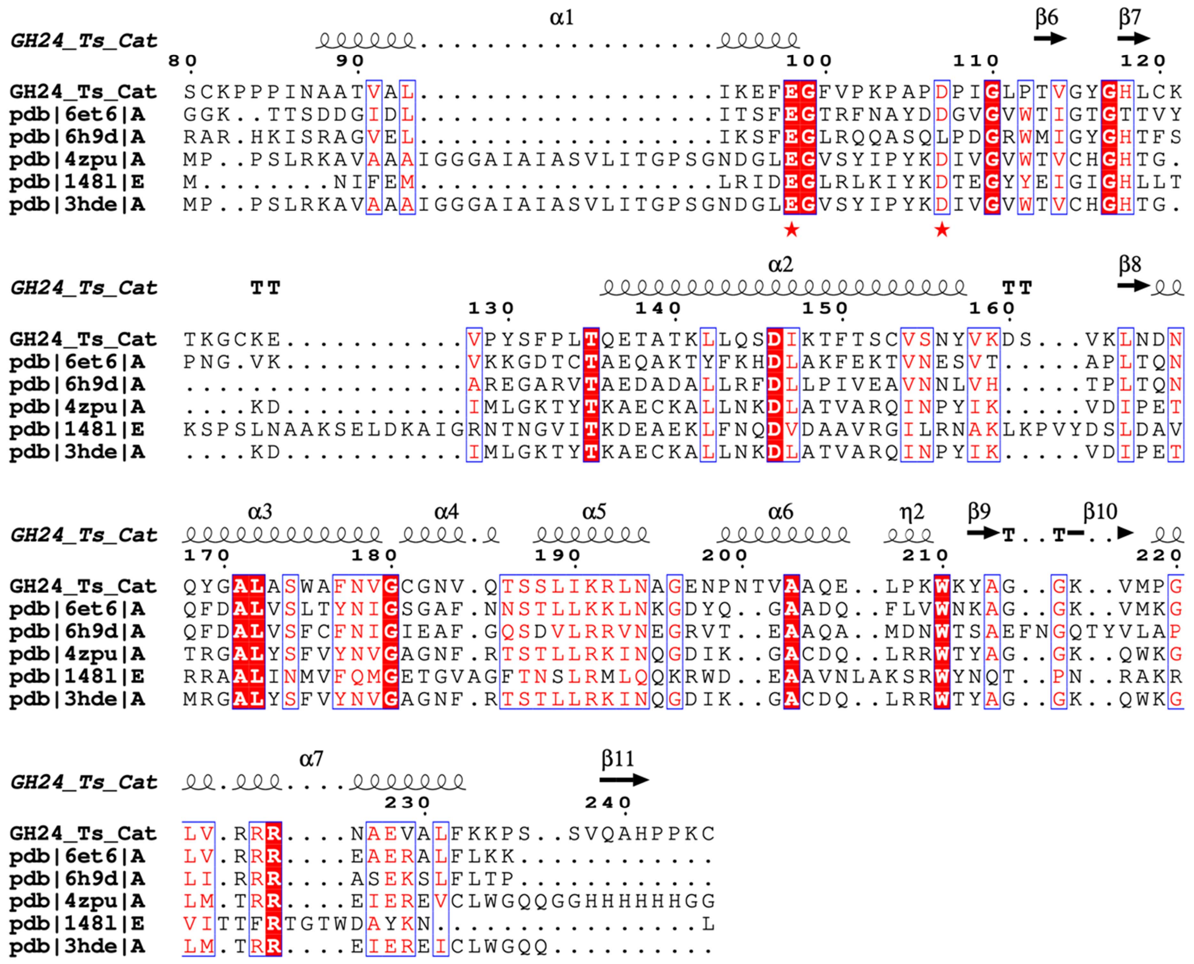

Figure S6 Sequences of a set of GH24 catalytic domains. These were aligned using T-coffee on the EBI server and displayed using ESPRIPT (Robert & Gouet, 2014). The secondary structure of the *Trichophaea saccata* enzyme is shown. The number of invariant residues is seen to be very few. The supposed catalytic Glu105 and Asp114 are indicated by an asterisk. The Asp is changed to a Leu in 6H9D – this is an inactive homologue.

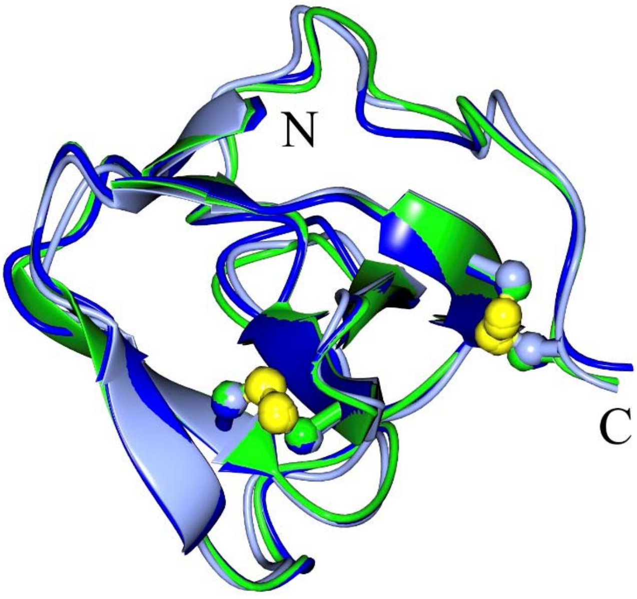

Figure S7 Superposition of two X-ray structures of SH3-like domains, from *Trichophaea saccata* (shown in grey) and *Penicillium virgatum* (in blue) muramidases and one SH3 domain from a model from AF2 database (A0A5M3Z971) for a protein from *Aspergillus terreus*. The SS-bridges are shown in ball and stick, the second SS-bridge stabilises the C-terminal loop, and possibly plays a role in forming the target-binding site

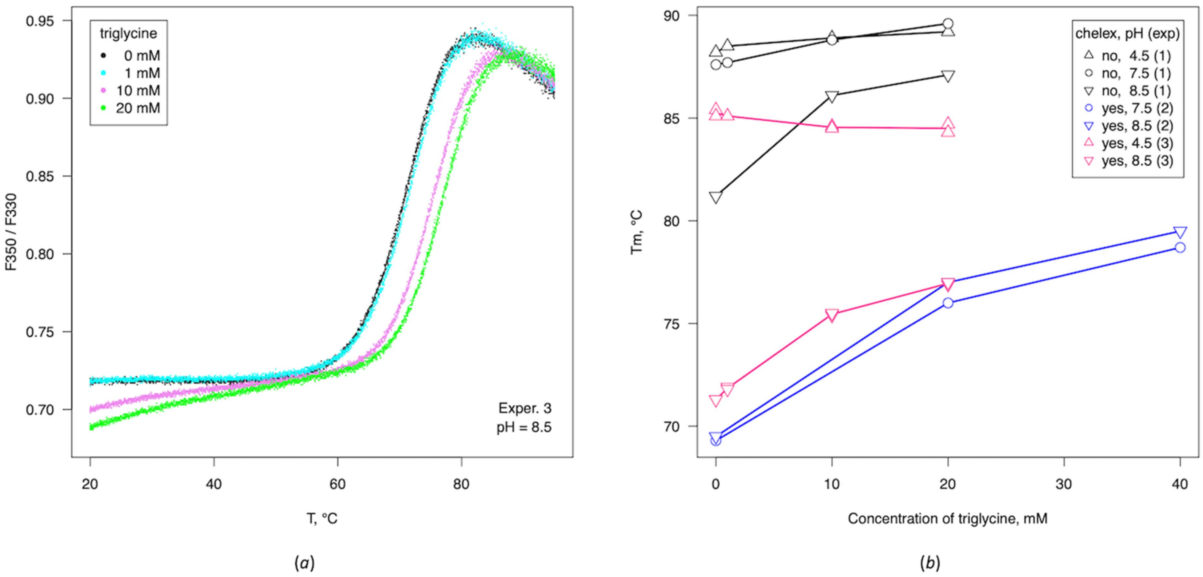

Figure S8 Thermal shift assay by nanoDSF for *Ts*CWBD – triglycine binding. Ligand binding increases protein stability at pH 8.5 for both Chelex-treated and untreated samples, indicating ligand binding at crystallisation conditions. The protein is highly stable at pH 4.5, and there is virtually no effect of ligand binding with or without chelex. At pH 7.5, a significant T_m shift is observed only for the Chelex-treated sample.

Altschul, S. F., Gish, W., Miller, W., Myers, E. W. & Lipman, D. J. (1990). *J Mol Biol* **215**, 403-410.

Dornberger-Schiff, K. & Grell-Niemann, H. (1961). *Acta Crystallographica* **14**, 167-177.

Kuroki, R., Weaver, L. H. & Matthews, B. W. (1993). *Science* **262**, 2030-2033.

Kuroki, R., Weaver, L. H. & Matthews, B. W. (1999). *Proc Natl Acad Sci U S A* **96**, 8949-8954.

Liu, Y., Li, M., Schnorr, K.M., Olsen, P.B. (2018). WO18206001.

Liu Y., S. K. M., Kiemer L., Skov L. K., Sandvang D. H., Cohn M. T., Li M. (2017). WO17000922.

Peng, Y., Leung, H. C. M., Yiu, S. M. & Chin, F. Y. L. (2010). *Lect N Bioinformat* **6044**, 426-440.

Robert, X. & Gouet, P. (2014). *Nucleic Acids Res* **42**, W320-324.

Ter-Hovhannisyan, V., Lomsadze, A., Chernoff, Y. O. & Borodovsky, M. (2008). *Genome Res* **18**, 1979-1990.
